# Spondyloarthritis, Acute Anterior Uveitis, and Fungi: Updating the Catterall–King Hypothesis

**DOI:** 10.3389/fmed.2018.00080

**Published:** 2018-04-05

**Authors:** Martin Laurence, Mark Asquith, James T. Rosenbaum

**Affiliations:** ^1^Shipshaw Labs, Montreal, QC, Canada; ^2^Division of Arthritis and Rheumatic Diseases, Oregon Health & Science University, Portland, OR, United States; ^3^Department of Ophthalmology, Oregon Health and Science University, Portland, OR, United States; ^4^Department of Medicine, Oregon Health and Science University, Portland, OR, United States; ^5^Department of Cell Biology, Oregon Health and Science University, Portland, OR, United States; ^6^Legacy Devers Eye Institute, Portland, OR, United States

**Keywords:** spondyloarthritis, acute anterior uveitis, reactive arthritis, ankylosing spondylitis, fungal infections

## Abstract

Spondyloarthritis is a common type of arthritis which affects mostly adults. It consists of idiopathic chronic inflammation of the spine, joints, eyes, skin, gut, and prostate. Inflammation is often asymptomatic, especially in the gut and prostate. The HLA-B*27 allele group, which presents intracellular peptides to CD8+ T cells, is by far the strongest risk factor for spondyloarthritis. The precise mechanisms and antigens remain unknown. In 1959, Catterall and King advanced a novel hypothesis explaining the etiology of spondyloarthritis: an as-yet-unrecognized sexually acquired microbe would be causing all spondyloarthritis types, including acute anterior uveitis. Recent studies suggest an unrecognized sexually acquired fungal infection may be involved in prostate cancer and perhaps multiple sclerosis. This warrants reanalyzing the Catterall–King hypothesis based on the current literature. In the last decade, many links between spondyloarthritis and fungal infections have been found. Antibodies against the fungal cell wall component mannan are elevated in spondyloarthritis. Functional polymorphisms in genes regulating the innate immune response against fungi have been associated with spondyloarthritis (*CARD9* and *IL23R*). Psoriasis and inflammatory bowel disease, two common comorbidities of spondyloarthritis, are both strongly associated with fungi. Evidence reviewed here lends credence to the Catterall–King hypothesis and implicates a common fungal etiology in prostate cancer, benign prostatic hyperplasia, multiple sclerosis, psoriasis, inflammatory bowel disease, and spondyloarthritis. However, the evidence available at this time is insufficient to definitely confirm this hypothesis. Future studies investigating the microbiome in relation to these conditions should screen specimens for fungi in addition to bacteria. Future clinical studies of spondyloarthritis should consider antifungals which are effective in psoriasis and multiple sclerosis, such as dimethyl fumarate and nystatin.

## Introduction

More than four decades ago, major histocompatibility complex (MHC) class I allele group HLA-B*27 was identified as a potent risk factor for developing spondyloarthritis (1, 2). Despite the strength of this association, mechanisms accounting for the link between HLA-B*27 and spondyloarthritis remain a mystery. The bacterial microbiome has attracted a great deal of attention as a possible explanation for this link (3). While we acknowledge the importance of bacteria, we describe a likely role for fungi and suggest that the prostate may be an important locale harboring microbes etiologically related to spondyloarthritis.

Recent studies have linked the immune response against fungi with prostate cancer (4) and multiple sclerosis (5). Sexual risk factors of prostate cancer (6–8) and multiple sclerosis (9–12) suggest that there may exist an as-yet-unrecognized sexually transmitted infection (STI) etiologically involved in these two diseases (4, 5, 13, 14). Epidemiological evidence suggests this elusive STI may be a fungal infection (15).

Many forms of spondyloarthritis have links with fungal infections, including ankylosing spondylitis (AS) (16), acute anterior uveitis (AAU) (16), Crohn’s disease (17, 18), and psoriasis (19, 20). One form of spondyloarthritis, reactive arthritis (ReA), has clear sexual risk factors—though causal associations with known STIs remain uncertain (21, 22). ReA and other spondyloarthritides are rare in children, and onset typically occurs in young adulthood (23, 24), mirroring the occurrence of STIs (25). In 1959, Catterall and King postulated a common sexually acquired infectious etiology for all spondyloarthritis types, whose primary focus in men is the prostate (26, 27).

In this article, we review the evidence which allowed Catterall and King to postulate their hypothesis, and related studies published in the six intervening decades. We then review the links between fungi and spondyloarthritis, in part to determine if the sexually acquired prostatic infection sought by Catterall and King (28) could be the same as the putative sexually acquired fungal infection suspected of causing prostate cancer (4, 15).

## Postulation of the Catterall–King Hypothesis

Between 1818 and 1948 many case reports were published describing a syndrome affecting men, consisting mainly of the simultaneous inflammation of the urethra, eyes, and joints (29). This syndrome was initially called Reiter’s disease. It is now called ReA due to the genital and enteric infections which often immediately precede onset. The classic eye inflammation in ReA is bilateral conjunctivitis on the external surface of the eye and less frequently unilateral AAU (30).

### Harkness’ Review of ReA (1949)

After studying 126 cases, Harkness published a comprehensive review of ReA (29). He made several key observations (Table 1). Harkness extended the definition of ReA to include incomplete cases in which either eye or joint inflammation was absent, and to similar cases in women where the main genital symptom was cervicitis. Harkness noted that generally no microbe could be observed or cultured to account for urethritis or arthritis symptoms, and that the presence of *Neisseria gonorrhoeae* in a subset of patients—previously thought to be causative—seemed to be coincidental. Some idiopathic urethritis cases in his series were likely caused by infections that were not as well characterized at the time, such as *Chlamydia trachomatis* (serology was positive in 15% of his cases) and Mycoplasmataceae species. Idiopathic urethritis remains frequent today: its prevalence is about 13% in healthy American men (31), and no infectious agent can be found in about half of American men presenting with urethritis at an STI clinic (32). Similarly, no causative microbe can be found in about half of sexually acquired ReA cases (22).

**Table 1:** Nine key observations in Harkness’ 1949 review of ReA.

Key observation	Confirmation
Sexual activity often immediately precedes ReA onset	Confirmed by many studies (33–35); enteric and idiopathic cases are also common (36–38)

Urethritis is abacterial (urethral cultures are mostly negative)	Confirmed by many studies (22, 31, 32), though sensitive universal microbiome assays have not been performed

Smears and cultures of synovial fluid/tissue are mostly negative	Confirmed by many studies (21, 36), though sensitive universal microbiome assays have not been performed

*Neisseria gonorrhoeae* is acting as a surrogate for sexual activity and is neither required nor causative	This is now generally accepted (30)

ReA symptoms may not all be present	Confirmed by Csonka (33, 39) and now generally accepted

ReA relapses are common	Confirmed by Csonka (33, 39) and now generally accepted

ReA occurs in women as well, but cervicitis (not urethritis) is the main genital symptom	This is now generally accepted (22)

When present, urethritis usually appears before other symptoms	This is now generally accepted (30, 40)

Sexually acquired ReA relapses can occur without sexual contact	Confirmed by Csonka (33, 39) and now generally accepted

*ReA, reactive arthritis*.

Harkness advanced a four-part hypothesis to explain the etiology of ReA: (a) a single as-yet-unrecognized infectious agent is necessary in all ReA cases; (b) some relapses are caused by non-sexual “additional factors” that reactivate the infectious agent; (c) the infectious agent cannot be cleared by the immune system in at least some cases and remains in a latent state between attacks; and (d) enteric microbes are not directly causative, but rather unleash the single causative infectious agent.

### Prostatic Inflammation in AS and ReA (1958)

The association between inflammation of the prostate—usually defined as a high concentration of leukocytes in expressed prostatic secretion obtained by prostate massage—and non-genital symptoms was firmly established by Visher in 1929 (41). Visher tested the expressed prostatic secretion of 500 consecutive young men admitted for any reason to the Veterans’ Hospital in Waukesha, Wisconsin (41). 87 men (17%) had a high prostate leukocyte concentration (41). Of these 87 men, 36 men had radiographs taken of their spine and sacroiliac joints, of which 20 showed signs of AS (55%) (41). This greatly exceeded the expected rate, though control radiographs were not used.

In 1958, a Swedish group (42) and a British group (43) published independent studies which replicated the association between prostate, spine, and sacroiliac inflammation reported by Visher. Both studies reported prostatic inflammation in a third of controls (22/66 and 28/85, respectively) and in nearly all AS patients (71/73 and 45/54, respectively) (42, 43). ReA cases were also included in these two studies, and a strong association with prostatic inflammation was found again (34/40 and 56/59, respectively) (42, 43). Many other studies reported high rates of prostatic inflammation in AS and ReA patients (44–47), though they did not include controls, so their results are difficult to interpret (48).

### The Catterall–King Hypothesis (1959)

By 1957, it was recognized that ReA often occurred in an incomplete form (29). King suspected that idiopathic AAU, a common comorbidity of ReA and AS, was part of the same syndrome even when it occurred alone. King thus predicted that prostatic inflammation would be associated with isolated AAU (49), as previously reported in small case series (50–54). To formally demonstrate this association, King asked Catterall to perform a prospective study of all new male uveitis cases from May 1957 to December 1958 at the Institute of Ophthalmology in London (49). Catterall’s study confirmed that AAU was strongly associated with prostatic inflammation as compared to controls and to other patients in the series (Table 2) (27, 28).

**Table 2:** Catterall’s prospective study of prostatic inflammation in male uveitis patients (27, 28).

Diagnosis	Symptoms suggestive of spondyloarthritis	Prostatic inflammation	Prostatic inflammation (combined)
AAU (isolated)	2 PF, 6 ASI	44/70 (63%)	107/133 (80%)
AAU and ReA	38 ReA	38/38 (100%)	
AAU and AS	25 AS	25/25 (100%)	

Chronic anterior uveitis	2 ReA, 1 ASI	8/19 (42%)	38/78 (49%)
Posterior uveitis	1 ReA	13/30 (43%)	
Generalized uveitis	1 AS, 4 ReA	17/29 (57%)	

No uveitis		1/15 (7%)	15/90 (17%)
Age matched controls		14/75 (19%)	

*AAU, acute anterior uveitis; PF, plantar fasciitis; ASI, atypical sacroiliitis; AS, ankylosing spondylitis; ReA, reactive arthritis; No uveitis, initial diagnosis was incorrect*.

Based on these prostatic inflammation association studies, Catterall and King advanced a three-part hypothesis (26, 28): (a) isolated AAU, ReA, and AS are part of the same syndrome; (b) an as-yet-unidentified genital infection is necessary for this syndrome; and (c) this infection is generally sexually acquired. The main marker of this genital infection in men was considered to be prostatic inflammation, as determined by counting the number of leukocytes in expressed prostatic secretion. Unfortunately, this was a low specificity marker because the vast majority of men with prostatic inflammation never develop the syndrome (43). This was thought to be due to rare genetic predispositions which were necessary for inflammation to occur outside the genital area (43). No assay available at the time managed to detect the putative infection (48). Four years later, one of King’s coworkers stated (55): “The trouble is that we have not yet identified the organism.”

### Replication by Dark and Morton (1968)

The association between prostatic inflammation and isolated AAU was not considered biologically plausible, and Catterall’s results were deemed unlikely (56, 57). All studies associating prostatic inflammation with isolated AAU, ReA, or AS used semi-quantitative leukocyte per high-powered field methods, which could have been biased by improper blinding during analysis or sample collection, yielding a spurious association.

To test the association between prostatic inflammation and isolated AAU with the least possible bias and highest accuracy, Dark and Morton used total ejaculate rather than expressed prostatic secretion (eliminating sample collection bias), used cell counting chambers rather than plain microscope slides (a fully quantitative counting method), blinded their analysis (eliminating observer bias), and excluded men with a history of urethritis or ReA (eliminating bias toward sexually acquired cases) (57). Their results were unequivocal: the association between genital inflammation and isolated AAU is real (57).

## HLA-B*27

The HLA-B*27 allele group is the strongest genetic risk factor for spondyloarthritis. Its association with AS exceeds odds ratios of 40 in Caucasian populations (58–60), and it is also strongly associated with ReA (36) and isolated AAU (61). Table 3 lists conditions associated with the HLA-B*27 allele group. Though hypotheses other than antigen presentation have been proposed, the association of *ERAP1* polymorphisms with AS in HLA-B*27 carriers strongly suggests major histocompatibility complex (MHC) class I antigen presentation to CD8+ T cells is part of the causative pathway leading to spondyloarthritis (62). The discovery of HLA-B*27 and its association with isolated AAU, ReA, and AS gave much credence to the Catterall–King hypothesis by confirming that these conditions shared an underlying immune mechanism (56). It also provided evidence that the putative etiological infectious agent was intracellular and that a genetically determined immune response caused symptoms (43).

**Table 3:** Conditions associated with HLA-B*27 spondyloarthritides.

Condition	Present in sexually acquired ReA		Present in AAU		Antibodies against fungi	*Candida*	*Malassezia*	*ERAP* alleles	*CARD9* alleles	*IL23R* alleles
Conjunctivitis	32%	(30)								

Uveitis (especially AAU)	8%	(30)			Mannan (16)					

Stomatitis	12%	(30)				+ (63)	+ (63)			

Cervicitis (women)	76%	(22)								

Cystitis	22%	(30)								

Prostatitis (men)	90%	(42, 43)	65%	(28, 57)						

NSU (men)	79%	(30)								

Balanitis circinata (men)	23%	(30)				+ (64)	+ (64)			

Keratoderma blennorrhagica	13%	(30)				– (65)	+ (65)			

Plantar fasciitis	20%	(30)	2%	(28)						

Peripheral arthritis	94%	(30)	29%	(28)	Mannan (16)					

Sacroiliitis	58%	(43, 47)	34%	(28, 57, 66)	Mannan (16)			rs30187 (60) rs2910686 (60)	rs1128905 (60)	rs11209026 (60)

Spondylitis										

Psoriasis					*M. furfur* (67, 68) *C. albicans* (67)	– (65)	+ (65)	rs27432 (69)		rs9988642 (69)

Crohn’s disease					Mannan (16, 70) Beta-glucan (70) Chitin (70)	+ (71)	+ (71)	rs2549794 (72) rs30187 (73)	rs4077515 (72)	rs11209026 (72)

Ulcerative colitis						+ (71)	+ (71)			

The antibodies against fungi column indicates which fungal antigens targeted by antibodies have been associated with the condition. Mannan is the mannose polymer coat of fungi targeted by ASCAs. The Candida and Malassezia columns indicate if these fungal genera are often present in affected sites in healthy adults. Associations with ERAP alleles suggest presentation of an intracellular peptide via MHC class I receptors affects disease risk. Associations with CARD9 and IL23R alleles suggest that the innate immune response against fungi affects disease risk. Prostatitis is defined as an elevated leukocyte concentration in expressed prostatic secretion. Symptoms strongly associated with HLA-B*27 frequently occur in non-HLA-B*27 carriers as well. The vast majority of psoriasis, Crohn’s disease, and ulcerative colitis cases occur in isolated form in non-HLA-B*27 carriers, so the link with these diseases is not very specific.

*ASCA, anti-Saccharomyces cerevisiae antibodies; AAU, acute anterior uveitis; MHC, major histocompatibility complex; ReA, reactive arthritis; NSU, non-specific urethritis; C. albicans, Candida albicans; M. furfur, Malassezia furfur*.

The expression of HLA-B in various tissues and cell types was recently measured by the Human Protein Atlas project (74). HLA-B exhibited highly variable expression in different tissues and surprisingly was either undetected or negligibly expressed in a number of tissues including hepatocytes, myocytes, and soft tissues. HLA-B was highly expressed in the secretory epithelial cells of the prostate (although less in the cervix), glandular cells of the small intestine and colon, as well as the skin. Its expression was not measured in the eyes or joints, though the strong expression of HLA-B*27 in synovial lining cells of AS patients was reported by an older study (75). HLA-B*27 presentation of intracellular antigens is thus consistent with HLA-B expression in all spondyloarthritis sites tested.

## The Usual Suspects

Enteric microbial epidemics and sexual risk factors have both been convincingly associated with the onset of ReA (76). Proving this link was relatively easy due to the short lag of a few weeks between enteric/genital symptoms and ReA symptoms. The list of confirmed or suspected precipitating infectious agents is very long, especially for enteric ReA (76–78). The relative incidence of enteric and genital ReA cases is not firmly established. The largest prospective community-based study, performed in Oslo (Norway) between 1988 and 1990, found that half of the cases were idiopathic and the remaining cases were evenly split between enteric and sexually acquired types (36). A smaller study published 10 years earlier reported a similar distribution (37). Another small study published 10 years later found a similar fraction of idiopathic cases, but a much lower fraction of sexually acquired cases as defined by *C. trachomatis* seropositivity (38).

Though various infections have been considered as possible triggers for isolated AAU and AS, fewer studies have investigated this link as compared to ReA. The onset of AS symptoms is gradual, so retracing infectious triggers which occurred years earlier is difficult.

### Enteric Infections in ReA

One of the best controlled studies of enteric ReA was conducted in 1962 on an American Navy ship after a sudden outbreak of *Shigella*-induced dysentery (40). Because the outbreak timing was circumscribed and the ship was at sea during the following months, all ReA cases coinciding with this outbreak could be traced (40). Out of a population of 1,276 male crew members, 602 proven cases of dysentery occurred, of which nine developed ReA (1.5%) (40). The sequence of symptoms varied between individuals, with six out of nine showing the complete triad and with urethritis preceding other symptoms in most cases (40). This matches ReA presentation described in sexually acquired cases (29, 33). Less well-controlled community-based outbreak studies also support enteric triggers for ReA, and have implicated many enteric infections beyond *Shigella* (76, 77). These infections include not only bacteria such as *Salmonella, Campylobacter, Yersinia, Clostridium difficile*, and *Escherichia coli* but also protists such as *Giardia lamblia* (79). Idiopathic enteric symptoms, where no plausible causative infection can be found, are also common in ReA (79). There is no obvious pattern linking these infections other than enteric inflammation.

### Genital Infections in ReA

Unlike enteric infections, genital infections like *Candida albicans* and STIs do not occur in large confined epidemics, making associations with ReA more difficult to prove. Prior to large prospective studies, sporadic ReA cases seemed concentrated in men who consulted for urethritis symptoms resembling gonorrhea and shortly thereafter developed inflammation of the eyes and joints (33). Sexually acquired ReA cases were thus initially (wrongly) deemed to be post-gonorrheal polyarthritis (47).

Sporadic ReA cases were mainly seen by urologists and venereologists, who considered urethritis a necessary symptom. In 1933, Harkness realized that a majority of urethral discharge cases were of non-gonococcal origin (80). The search for genital infections which could explain idiopathic urethritis and ReA began in earnest after World War II, and the presence of *C. trachomatis* (29), *Ureaplasma urealyticum* (29), *Mycoplasma hominis* (29), and *Trichomonas vaginalis* (26) was quickly demonstrated in some cases. However, even after accounting for these new genital infections, most urethritis cases remain unexplained (22, 31, 32).

*C. trachomatis* became widely recognized as an STI in the 1970s (81) and is currently the genital infection most convincingly associated with ReA (21, 82). The strongest evidence of such an association can be found in case–control studies that measure either serological markers of past exposure to *C. trachomatis* or the presence of *C. trachomatis* itself in the genital tract (22, 36, 37, 83). The largest prospective community-based study cultured *C. trachomatis* in 25 of 112 ReA cases (22%) which occurred in Oslo (Norway) between 1988 and 1990 (36). The largest serological study analyzed 323 ReA cases referred to the Diagnostic-Research Centre of Sexually Transmitted Diseases in Bialystok (Poland) between 2001 and 2012, finding *C. trachomatis* IgG seropositivity in 70 cases (22%) (83). Both studies considered these rates to be much higher than those of control populations.

Due to the lack of natural experiments such as confined epidemics, it is difficult to demonstrate that *C. trachomatis* directly causes a subset of ReA cases and is not acting as a surrogate for another genital infection—as occurred with *N. gonorrhoeae* before it. This was well understood in 1968, when early reports of *C. trachomatis* in ReA were met with skepticism because *C. trachomatis* rates in ReA series were similar to those seen in STI clinics (84), suggesting it was acting as a surrogate for sexual activity and was not directly involved (85). Exposure to *C. trachomatis* cannot be demonstrated in a majority of sexually acquired cases (22, 82), let alone in all types of ReA cases combined (36, 38, 83), and two prospective STI clinic studies have confirmed that *C. trachomatis* is not associated with ReA in high STI risk populations (86, 87).

*N. gonorrhoeae* and *C. trachomatis* can be excluded as a cause of most ReA cases, even in STI clinic series (22, 29, 82, 84, 86, 87). Their association with ReA could be due to these two STIs acting as surrogates for an as-yet-unrecognized sexually acquired infection, as proposed by Catterall and King, or it could be due to a direct etiological role in a minority of sexually acquired ReA cases. *N. gonorrhoeae* and *C. trachomatis* are both associated with recent changes in sexual partners (88) and thus to genital exposure to a new set of microbes. In contrast with other STIs, their clearance by the immune system within a few months (89) makes them excellent markers of a recent change in sexual partners. If known STIs are acting as surrogates for an as-yet-unrecognized sexually acquired infection which causes ReA, then the strongest associations would be expected to be found with *N. gonorrhoeae* and *C. trachomatis*.

### Infections in AS and AAU

Infectious triggers of AS and AAU have not been as widely studied as in ReA. The studies which have been run are small. Demonstrating associations with infections occurring a month before ReA onset is easy in comparison, whereas it often takes a decade for AS to be recognized (24): triggering infections will be hard to identify because causative microbes may have been cleared and seropositivity may have been lost in the interim. For example, *C. trachomatis* IgG seropositivity is lost within 6 years when using microimmunofluorescence assays (90).

Antibodies against *Klebsiella* have been associated with AS in many studies (91), and *Klebsiella* stool cultures have been associated with disease activity in AS (92) and AAU (93), though *Klebsiella* cannot be found in most patients (94). Antibodies against peptidoglycan, a common component of bacterial cell walls, have been associated with AS (95) and other spondyloarthritis types (96).

Two small studies found high rates of *C. trachomatis* in male AS patients, respectively, using cell culture (15/31) (97) and IgG/IgA solid phase enzyme immunoassay (20/32) (98). The selective reporting of *C. trachomatis* in only a subset of AS patients in the first study and the lack of comparison to a control group in both studies means these results are difficult to interpret. A study of genital infections in women with AS reported a similarly high *C. trachomatis* rate detected by cell culture (15/32), which was significantly higher than the rate measured in controls (5/33) (99). However, a similar study performed in men with AS using sensitive molecular methods found much lower rates of *C. trachomatis* (1/32), which were indistinguishable from controls (3/120) (98); note that this study found high rates of *C. trachomatis* antibodies in these AS patients (20/32) (98), as reported earlier in this paragraph. Finally, two small *C. trachomatis* serological studies of AS patients of both sexes did not find a statistically significant association, perhaps due to low power (100, 101).

Studies analyzing spondyloarthritis phenotypes reported an increased risk of AAU in *C. trachomatis* seropositive patients (OR = 7.0, 95% CI: 1.1–44.1) (98) and *Saccharomyces cerevisiae* seropositive patients (OR = 4.36, 95% CI: 1.08–17.64) (16). Anti-*Saccharomyces cerevisiae* antibodies *(*ASCAs) bind to the mannose polymer (mannan) coat of all fungi: such antibodies can be generated in response to any fungal infection. ASCAs were also associated with peripheral arthritis (OR = 3.78, 95% CI: 1.57–9.15) (16) and inflammatory bowel disease (OR = 3.43, 95% CI: 1.15–10.20) (16). Spondyloarthritis and AS have been linked to ASCAs in many studies (16, 102–105), though the association is not as strong as in Crohn’s disease (70, 106).

Two small serological studies did not find an association between AAU and *C. trachomatis* when comparing with age-matched controls, nor did they find an association with any enteric infection suspected of causing ReA (107, 108). Neither study measured ASCAs.

Strong consistent associations between AS/AAU and infections suspected of causing ReA are lacking. The moderate association between STI seropositivity and AS/AAU implied by the Catterall–King hypothesis cannot be confirmed nor refuted by current studies: higher powered studies are warranted.

## Age at Onset

Ankylosing spondylitis is more common and has a more predictable course than ReA and AAU, making it easier to study from an epidemiological point of view. ReA and AAU typically occur in attacks lasting a few weeks or months, whereas AS is characterized by long-term inflammation of the sacroiliac joints and spine.

### Age at Onset of AS

Ankylosing spondylitis risk before puberty is very low (23, 24). Its risk of onset increases sharply around the age of 15 years, peaks during young adulthood (age: 18–29 years), and tapers off exponentially over the following 30 years (23, 24). This distribution suggests environmental factors necessary for AS (if any) affect mainly adults, not children.

The observation that many HLA-B*27 carriers do not develop AS suggests that additional genetic and environmental factors contribute to the disease. AS monozygotic twin concordance does not reach 100% (109), strongly supporting the existence of environmental factors. Because the monozygotic twin concordance observed in AS exceeds 50% (109), environmental factors required for triggering the disease in genetically susceptible individuals must be ubiquitous (110). This is also consistent with the tapering off of AS onset risk after the age of 30 years, since by that age most individuals would presumably have been exposed to any environmental trigger and have already developed symptoms.

Sexually acquired infections match well with the age at onset of AS because their incidence is very low in children and peaks in young adulthood (25). However, no known sexually acquired infections other than all human papillomavirus (HPV) types combined are present in over half of the population. For STIs which can be cleared by the immune system, peak prevalence occurs between 18 and 24 years of age (89). STI prevalence in Americans of this age group was estimated to be 53.8% for HPV (all types), 3.9% for herpes simplex virus type 2, 1.6% for *C. trachomatis*, 0.9% for *T. vaginalis*, and 0.3% for *N. gonorrhoeae* (89). *C. trachomatis* and *N. gonorrhoeae* are both well-established risk factors of ReA; other STIs in this list have not been widely studied in ReA because they do not often cause urethritis. Lifetime risk of sexual exposure to HPV (all types) in men is estimated to be 91% (111), so this infection reaches a high enough fraction of the population to be able to cause AS through a hit-and-run mechanism. In contrast, it appears unlikely that well over half the male population could be exposed to either *C. trachomatis* or *N. gonorrhoeae*, though formally demonstrating this is difficult based on currently published studies (89).

### Age at Onset of ReA and AAU

Studies of the age at first attack of ReA and AAU are much smaller than those related to AS. AAU is rare in children, and the highest risk of onset occurs in young adults (66, 112, 113). Sexually acquired ReA generally does not occur in children due to a lack of sexual activity, and its rate peaks in young adults (34, 35, 114); this distribution is very similar to that of AS. Enteric ReA has a more even age distribution within adults (36) and is also rare in children (115–117). At first blush, the paucity of enteric ReA cases in children is difficult to explain.

The largest sexually acquired ReA cohort study demonstrated that relapses sometimes coincide with genital or enteric infections but in many cases seem unprovoked (33, 39). Most of these relapses were attributed to flare-ups rather than to repeated exposure to triggering microbes (39). Urethritis is often the first symptom in enteric ReA, suggesting enteric infections are acting as one of many possible cofactors that can trigger flare-ups of a latent genital infection. This could explain why a wide range of enteric infections are associated with ReA, and why enteric ReA is rare in children (115–117) despite ample exposure to enteric microbes (115).

## Links with Fungi

The association between ASCAs and spondyloarthritis (16, 102–105) warrants analyzing links between fungal infections and conditions listed in Table 3. In a recent study, Maillet et al demonstrated that ASCAs are more strongly associated with peripheral symptoms (uveitis, arthritis, and inflammatory bowel disease) than axial symptoms (spondylitis and sacroiliitis), and with the absence of HLA-B*27 alleles (16). ASCAs are a biomarker of CD4+ T cell recognition of fungal mannoproteins, suggesting the recognition of fungi by CD4+ T cells may play an important role in peripheral spondyloarthritis symptoms, and in cases where antigen presentation to CD8+ T cells is less efficient due to the absence of HLA-B*27 alleles. CD4+ T cell recognition of *Malassezia* antigens resulting in a Th1 response has been reported in psoriasis (20), though it has not been studied in spondyloarthritis.

Rare homozygous mutations in either *CARD9* or in the IL-17/IL-23 pathway cause chronic mucocutaneous candidiasis by impairing the immune response against fungi (118–120). Similarly, IL-17 inhibitors increase candidiasis risk (121). Genome wide association studies have linked prevalent functional *CARD9* and *IL23R* polymorphisms to AS and associated conditions (Table 3). These genes are involved in the inflammatory cascade downstream of phagocyte recognition of fungal cell wall components beta-glucan (mainly through Dectin-1) and mannan (mainly through Dectin-2) (110, 122, 123). These are the two primary antigens leading to phagocytosis of fungi such as *Malassezia furfur* (124) and *Cryptococcus neoformans* (125). These links with fungi suggest that the immune response against fungal antigens may be an important component of spondyloarthritis, and interventions targeting cytokines associated with fungal infections—for example, the anti-IL-17A drug secukinumab (126)—may improve symptoms.

### Fungi and Uveitis

ASCAs are strongly associated with uveitis in spondyloarthritis patients (16), but antibodies against various bacteria suspected of causing ReA are not (107, 108). Similarly, circulating CD4+ T cells in uveitis patients are more sensitive than controls to fungal antigens (*C. albicans*) after a 24-h antigen exposure period (127), whereas sensitivity to bacterial antigens (*Staphylococcus aureus, Clostridium tetani*, and *Mycobacterium tuberculosis*) and protist antigens (*Toxoplasma gondii*) are similar in both groups (127). Intermediate uveitis is associated with multiple sclerosis onset (128) and with the HLA-DRB1*1501 allele (129), which are both associated with the immune response to fungi (5). A small study reported that oral dimethyl fumarate, a fungicidal compound known to be effective in multiple sclerosis and psoriasis, improved chronic idiopathic uveitis symptoms in four patients (130).

Though most cases of uveitis are considered idiopathic, some cases can be attributed to infections: bacteria (especially *M. tuberculosis, C. trachomatis, Treponema pallidum*, and *Borrelia burgdorferi*), viruses (especially herpes viruses), protists (especially *T. gondii*), and fungi (especially *C. albicans*) can cause uveitis (131). In a very large study of uveitis etiology, infections were reported in 13% of anterior uveitis, 7% of intermediate uveitis, 40% of posterior uveitis, and 43% of panuveitis cases (131). The abovementioned studies indicate that increased immune sensitivity to fungal antigens may be an important risk factor of idiopathic uveitis, especially in association with spondyloarthritis and multiple sclerosis.

### Fungi and Inflammatory Bowel Disease

About 10% of spondyloarthritis patients also have inflammatory bowel disease (Crohn’s disease or ulcerative colitis) (16, 24), a rate significantly higher than that of the general population (<1%) (132). Enteric inflammation can be found in about 60% of spondyloarthritis patients, though it is usually subclinical (133). Functional *CARD9* and *IL23R* alleles are also associated with isolated Crohn’s disease and ulcerative colitis (Table 3), suggesting the immune response against fungi may play an important role here as well.

The recognition of fungal antigens is accentuated in human peripheral blood mononuclear cells (PBMC) from Crohn’s disease patients as compared to controls (134, 135); this was attributed to increased expression of Dectin-1, Dectin-2, and the mannose receptor (135). A similar study focused on bacterial antigens found that CD4+ T cell activation through antigen presentation by PBMC was elevated in inflammatory bowel disease patients as compared to controls when exposed to either *C. albicans* or nine bacterial species (136). Because CD4+ T cells recognize antigenic peptides which are more species specific than sugars recognized by monocytes, this study should be repeated with a wider panel of medically important fungi including *Malassezia* and *Cryptococcus* species. These two genera are taxonomically very distant from *Candida* and are expected to share few antigenic peptides despite sharing antigenic sugars such as beta-glucan and mannan. CD4+ T cell recognition of *Candida* and *Malassezia* antigens can be very different, as demonstrated in psoriasis (20). Crohn’s disease is strongly associated with antibodies against conserved fungal antigen sugars (especially mannan, but also beta-glucan and chitin) (70), and recent gut microbiome studies suggest a fungal etiology (17, 18, 137, 138).

Three recent studies using vedolizumab in Crohn’s disease and ulcerative colitis patients reported that this intervention triggered peripheral arthritis, sacroiliitis, or psoriasis in about 10% of cases (139–141); these are the most common symptoms associated with spondyloarthritis (Table 3). Vedolizumab blocks the α4β7 integrin homing receptor, preventing lymphocytes originating from the gut from returning there during recirculation (141). It is thus plausible that following activation due to antigen exposure in the gut, stray lymphocytes subsequently trigger inflammation upon reaching the peripheral joints, sacroiliac joints, and skin (142, 143) by recognizing a similar or identical antigen in these sites (144). Since Crohn’s disease is strongly associated with an immune response against fungi, these may be fungal antigens.

The distribution of the age at onset of isolated AS (24) is very similar to that of AS with inflammatory bowel disease (24) and to that of isolated Crohn’s disease (145, 146): risk is low before puberty, increases sharply around the age of 15 years, peaks during young adulthood (age: 18–29 years), and tapers off over the following 30 years (146). Though Crohn’s disease is not as strongly associated with an MHC class I allele as AS, associations with *ERAP* alleles and interactions between *ERAP* alleles and MHC class I alleles have also been reported in Crohn’s disease (72, 73). It is thus plausible that exposure to the same sexually acquired intracellular fungal infection is necessary for both spondyloarthritis and isolated Crohn’s disease, and that genetic predisposition determines which symptoms appear following exposure to this environmental factor.

### Fungi and Psoriasis

Approximately 15–35% of spondyloarthritis patients also have psoriasis (16, 24), which is somewhat higher than the rate measured in the general population (<10%) (147). Many studies have associated psoriasis with fungi. *Candida* colonization of the gut (19, 148) and antibodies against *C. albicans* (67) and *M. furfur* (67, 68) are associated with psoriasis. The application of lysed *M. furfur* cells to the skin of psoriasis patients induces psoriasis-like lesions much more frequently than in controls (149). Psoriasis patients’ PBMC readily produce a Th1 response when incubated with *M. furfur* antigens (20), whereas the same response does not occur in controls, nor when using *C. albicans* or *Trichophyton rubrum* antigens (20). The chitin binding protein YLK-40 is associated with psoriasis and is a marker of disease severity (150); chitin is a highly conserved fungal antigen which is not present in bacteria or in human cells. Finally, many fungicidal compounds have been shown to reduce psoriasis symptoms (151–158), though such drugs can also induce psoriasis flare-ups (159)—such flare-ups have been tentatively attributed to a Jarisch–Herxheimer reaction (154, 156).

Though the incidence of psoriasis peaks at about the same age as AS (24), incidence in children and older adults is much higher in psoriasis (160). The age at onset of isolated AS and AS with psoriasis is nearly identical (24). This suggests that psoriasis may be a heterogeneous disease, of which only a subset of cases share a common etiology with AS (161). A recent study of *ERAP* alleles in psoriasis supports this hypothesis: *ERAP* alleles are only associated with psoriasis onset in adolescents and young adults (162), suggesting that the same sexually acquired intracellular fungal infection may be necessary for both spondyloarthritis and this subset of psoriasis cases.

The efficacy of oral nystatin in reducing psoriasis symptoms (154–157) [oral nystatin is not absorbed and thus limited to killing fungi in the gut (163, 164)], the expression of αEβ7 integrin by CD8+ T cells in psoriatic lesions (165) (αEβ7 integrin is a marker of mucosal origin), and the triggering of psoriasis by vedolizumab (140, 141) suggest that some psoriasis cases may be caused by lymphocytes originating in the gut. The many links with fungi described earlier and the associations between *ERAP* alleles and psoriasis (162) suggest that similar intracellular fungal antigens are present in the gut and on the skin, providing a common antigenic target for CD8+ T cells migrating from the gut to the skin. The only fungal genus currently known to be highly prevalent on the human skin is *Malassezia* (65), present in both healthy skin and psoriatic lesions (166). Several groups have proposed that a loss of immune tolerance to *Malassezia* may cause some psoriasis cases (149, 151, 153, 167, 168). *Malassezia* are found within skin keratinocytes (169) and have recently been detected in the gut (71, 138, 170).

### Fungi and Prostate Disease

The presence of idiopathic prostatic inflammation affecting men has been recognized for a very long time, both in isolation and in association with spondyloarthritis (41, 43, 48). The association between prostatic inflammation and spondyloarthritis, combined with other genital symptoms and sexual risk factors in ReA, suggests that a chronic genital infection may reach the prostate and cause these conditions (26, 27).

Fungal infections of the prostate are considered rare in immune-competent individuals (171), and few studies have investigated possible fungal etiologies in prostate disease (4). The recent discovery of an abundant fungicidal protein in the prostate (172) indicates that a fungal infection reaches this site, hence necessitating such an antimicrobial protein. This protein is called either prostate secretory protein 94 (PSP94) or beta-microseminoprotein, and is encoded by the *MSMB* gene. High PSP94 concentration in the prostate protects men from prostate cancer in a dose-dependent manner (173–176), suggesting prostatic fungi may be an important etiological component of prostate cancer (4). PSP94 concentration interacts with sexual risk factors in affecting prostate cancer risk, suggesting this fungal infection may be sexually acquired (15). Sexual risk factors have been widely studied in prostate cancer and are well recognized (6–8), though no specific causative infection has been strongly associated with prostate cancer (177). Due to inhibition by calcium ions (172), PSP94’s fungicidal activity within the prostate is restricted to the cytosol of secretory epithelial cells, suggesting that targeted fungi must be intracellular and must invade these cells (4).

Interestingly, an aberrant truncated form of PSP94 lacking the fungicidal region is strongly associated with benign prostatic hyperplasia (BPH) (178, 179), and prostate epithelial cells in BPH tissue do not stain for PSP94 in histological studies, as opposed to healthy tissue (180, 181). This suggests that the cytotoxic T cell immune response against secretory epithelial cells observed in BPH (182) may be triggered by the loss of PSP94-mediated immunity against a ubiquitous (183) intracellular fungal infection.

Histological studies have found melanin inclusions within secretory epithelial cells of the prostate targeted by CD8+ T cells (184–186), though a fungal infection was not considered as a possible explanation. Fungi synthesize melanin as a defense mechanism when exposed to fungicides (187, 188), so an intracellular fungus exposed to PSP94 within these cells could explain the presence of melanin.

PSP94 is also present in glandular epithelial cells of the cervix (189) [the main genital inflammation site of ReA in women (22)] and colon (189, 190) (one of the main inflammatory bowel disease inflammation sites). It is not present in the skin (190). Its presence in the eyes and joints has not been tested.

PSP94’s ortholog in pigs has conserved its fungicidal activity (172) despite rapid evolution (191) resulting in changes to half of the amino acids in the protein (192). Humans and pigs shared an ancestor about 90 million years ago, suggesting that PSP94 is coevolving with fungal infections in both species, and PSP94’s fungicidal activity confers a selective advantage to host fitness (193).

### Animal Models

PSP94’s conserved fungicidal properties suggest that rodents may also be infected with fungal species targeted by this protein. In 1956, Pearson noticed that the injection of Freund’s adjuvant (lysed *Mycobacterium* suspended in oil and water) into the footpad of Wistar and Long-Evans rats resulted in symptoms resembling ReA: lymphocyte-mediated inflammation of the joints, genitals, skin, and eyes ensued 10–16 days later (194, 195). Lipidic *Mycobacterium* extracts readily induced arthritis as well, suggesting that the adjuvant’s causative antigens were not proteins or peptides (195). It was later shown that alpha beta T cells were the main mediators of inflammation (196). Because alpha beta T cells directly recognize peptides rather than lipids, offending antigens may have already been present at the site of adjuvant administration. Host and commensal microbe antigens were considered the most probable target of these T cells, both in the footpad and in the many other sites of inflammation (195). Such sites appeared to be sterile by cell culture (195), and no self-antigens were strongly associated with this condition (197): the underlying mechanisms in this animal model remain unresolved (3). Likewise, the antigenic target in the widely used HLA-B27/β2m transgenic rat model of spondyloarthritis remains to be identified. It has been demonstrated that development of genital tract inflammation precedes and is required for the development of arthritic symptoms in these animals (198). We therefore hypothesize that a fungal infection at this site could be a contributing agent to the ensuing pathology.

Reactive arthritis-like symptoms can be induced in genetically predisposed rodents through exposure to various fungal stimuli: *C. albicans* (199), beta-glucan (110, 200), and mannan (110, 123). Dectin-2, CARD9, and IL-23 seem to be important in these animal models because knocking out Dectin-2 or CARD9 or blocking IL-23 reduces symptoms (110, 123).

An animal model of arthritis based on the injection of *Streptococcus pyogenes* cell wall antigens into the knee joint of mice demonstrated that a minute quantity of *C. albicans* added to this injection skewed the T cell immune response toward Th17, markedly increasing the level of inflammation (201). Interestingly, *C. albicans* alone, even in large quantities, caused little inflammation (201). This suggests that the immune response to fungal infections is muted unless inflammatory cofactors such as bacterial antigens are also present, giving a plausible explanation for Pearson’s animal model of ReA: *Mycobacterium* antigens may have stimulated an immune response against an elusive fungal infection already present in the rat footpad and other sites of inflammation. For example, *Mycobacterium* glycolipid trehalose dimycolate upregulates the expression of Mincle (202), and Mincle was recently shown to be a key receptor in an animal model of *Mycobacterium*-induced uveitis (203). Mincle has a very high affinity to alpha-mannose, an antigen found in *Malassezia* but not in other medically important fungal species (204). A very recent study reported that alpha-mannan induces uveitis in an animal model with an efficiency similar to *Mycobacterium* antigens (123). These findings suggest antigenic challenges may increase phagocytic activity directed against *Malassezia*, leading to activation of alpha beta T cells which recognize *Malassezia* peptides.

The hypothesis of an adaptive immune response against a fungal infection proposed in the previous paragraph is quite speculative and would be on more solid ground if there existed a disseminated ubiquitous fungal infection in an animal. One such infection was discovered in 2012 in mealworms (205): this fungus-like eukaryote is vertically transmitted to all individuals and is present throughout the mealworm body, though it is concentrated in the genitals and is sexually transmitted as well (205). To the best of our knowledge, no fungal infection with similar properties has been found in a rodent or other mammal. If such a fungal infection existed in humans, it could explain why a heterogeneous set of inflammatory stimuli can cause ReA: each stimulus can trigger the loss of immune tolerance to this disseminated fungal infection by acting as an immunological adjuvant which provokes an adaptive immune response against fungal antigens. This would explain why fungal colonization and fungal antigens are effective at breaking immune tolerance. If this elusive fungal infection was mainly sexually acquired in humans, this would explain why children and young teens are at very low risk of conditions associated with HLA-B*27, and why ReA has manifest sexual risk factors and genital symptoms. Finally, a fungal etiology would explain why functional polymorphisms in genes which are part of the immune response against fungi are associated with conditions listed in Table 3.

## Conclusion

The hypotheses put forward by Harkness in 1949 to explain the etiology of ReA (29), and expanded by Catterall and King in 1959 to include prostatic inflammation, AS, and isolated AAU (26, 28), have remained consistent with the scientific literature published since. The evidence reviewed here supports the existence of an as-yet-unrecognized genital infection which is a necessary etiological factor in spondyloarthritis. While the existence of an as-yet-unrecognized genital infection may seem to be far-fetched at first, such a hypothesis has been proposed to explain the sexual risk factors of multiple sclerosis (9, 14) and prostate cancer (7, 8, 13) based on evidence completely unrelated to spondyloarthritis (Table 4). Recent studies have implicated fungal infections in multiple sclerosis (5, 206–208) and prostate cancer (4, 15, 172), and these infections appear to be intracellular in both cases (Table 4) (4, 209, 210).

**Table 4:** Main observations supporting a common sexually acquired intracellular fungal infection in spondyloarthritis, prostate disease, and multiple sclerosis.

	Spondyloarthritis, reviewed here	Prostate disease, reviewed in Ref. (4)	Multiple sclerosis, reviewed in Ref. (5)
Sexually acquired	Sexual risk factors of reactive arthritis (especially *Neisseria gonorrhoeae* and *Chlamydia trachomatis*) Paucity of spondyloarthritis before the age of 15 years, peak onset during young adulthood (age: 18–29 years) Genital involvement (especially prostatitis and urethritis)	Sexual risk factors of prostate cancer (age at first intercourse, number of sexual partners, and exposure to any STI)	Sexual risk factors of multiple sclerosis (especially herpes simplex virus type 2) Paucity of multiple sclerosis before the age of 15 years, peak onset during young adulthood (age: 18–29 years)

Fungal	Antibodies against fungi associated with spondyloarthritis and Crohn’s disease *CARD9* and *IL23* alleles suggest that detection of fungal antigens is occurring in spondyloarthritis, Crohn’s disease and ulcerative colitis PBMCs more sensitive to fungal antigens in Crohn’s disease and uveitis. *Malassezia* strongly associated with granulomatous pediatric Crohn’s disease *Malassezia* patch test induces psoriatic inflammation PBMC Th1 response to *Malassezia* strongly associated with psoriasis Enteric *Candida* colonization associated with psoriasis Fungicides reduce psoriasis and psoriatic arthritis symptoms	PSP94 protects men from prostate cancer in a dose-dependent manner and is an antimicrobial protein targeting fungi (not bacteria) PSP94 truncation is a biomarker of BPH Melanin can be found in the prostate	Antibodies against fungi associated with multiple sclerosis HLA-DRB1*1501 increases risk of multiple sclerosis and causes excessive immune response against fungi Fungicides reduce multiple sclerosis symptoms

Intracellular	HLA-B*27 and *ERAP1* alleles suggest that intracellular antigen presentation is occurring	PSP94 only fungicidal within cytosol of prostate secretory epithelial cells (elsewhere in the prostate, it is inhibited by calcium ions) PSP94 truncation in BPH coincides with cytotoxic T cell response Prostate secretory epithelial cells containing melanin inclusions are targeted by CD8+ T cells	CD8+ T cells in multiple sclerosis lesions and *ERAP1* alleles suggest that intracellular antigen presentation is occurring

*BPH, benign prostatic hyperplasia; PBMC, peripheral blood mononuclear cells; PSP94, prostate secretory protein 94; STI, sexually transmitted infection*.

A commonly held view is that ReA-causing inflammation is aseptic, and chronic inflammation in the joints, spine, and eyes is due to autoimmunity (75). Associations with *ERAP* alleles and the wide variety of MHC class I alleles present in HLA-B*27-negative patients suffering from conditions listed in Table 3 suggest that many epitopes can be involved in the disease process. Thus, the hypothesis of molecular mimicry-induced autoimmunity triggered by an intracellular infection confined to the genitals cannot easily explain non-genital symptoms, unless this infection mimicked a wide range of human antigens (this seems unlikely). This suggests that the putative infection is not confined to the genitals, but rather spreads throughout the body and provides antigens necessary for inflammation in all affected sites, as proposed by Visher (41). This hypothesis is plausible because the only known microbial STI in humans which cannot be cleared by the immune system also spreads from the genitals and causes a wide variety of seemingly unrelated symptoms in infected sites: these symptoms are known as secondary and tertiary syphilis, and are caused by the bacterium *T. pallidum* (211).

**Figure 1:**
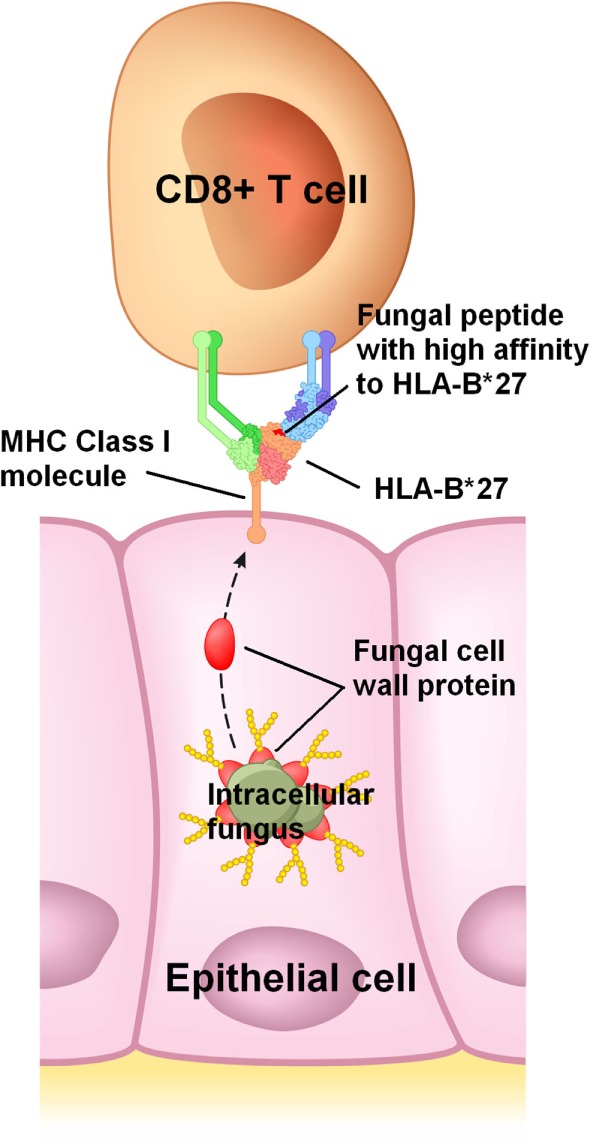
Proposed mechanism for HLA-B*27 in spondyloarthritis. HLA-B*27 would efficiently bind to a peptide from an abundant protein present in or on an intracellular fungus, and then present this peptide to CD8+ T cells on the infected host cell’s surface. In this example, a peptide from a fungal cell wall mannoprotein is presented to a CD8+ T cell. Cell wall mannoproteins are good antigen candidates due to their abundance, though presentation of peptides from other fungal proteins is also plausible.

The many links between fungi and conditions listed in Table 3 reviewed here suggest that a ubiquitous intracellular fungus, which usually reaches the genitals of adolescents and young adults through sexual activity, is a necessary etiological factor in these conditions. In a majority of individuals, this infection would remain asymptomatic or subclinical because immune tolerance is maintained. How and where immune tolerance is lost would be mostly genetically determined (43); this would explain why symptoms vary widely between infected individuals and why different conditions in Table 3 are associated with different genes. For example, HLA-B*27 molecules would have a high affinity to an antigenic protein in or on this fungus (Figure 1), frequently leading to a loss of immune tolerance in the sacroiliac joints and spine through the detection of this fungus in these sites. When immune tolerance is marginal, extraneous factors such as unrelated enteric or genital microbes would cause exacerbation of symptoms or flare-ups by acting as immunological adjuvants. These adjuvants are likely bacteria and unrelated fungi which infect or colonize mucosal surfaces, pushing lymphocytes in these sites to recognize the intracellular fungus, clonally expand, recirculate, enter non-mucosal tissue, and cause inflammation by detecting the intracellular fungus in such tissue.

The updated Catterall–King hypothesis proposed here has important implications for future studies. The microbiome of the prostate and cervix should be included in studies of conditions listed in Table 3. The many links with fungal infections described here highlight the limitations of 16S consensus microbiome techniques which can only detect bacteria. Techniques such as deep sequencing of total RNA (212–214) or DNA (166), while more expensive, should be used because they can detect microbes from the entire tree of life (including fungi). Recent studies have shown that many as-yet-unrecognized infections are present in humans (166, 215), so the existence of a novel microbe present in the genitals and in other sites is plausible. For example, molecular studies of oral (216) and genital (217) fungi in healthy individuals found many novel species. It would be interesting to know if fungal colonization of the gut is a risk factor of spondyloarthritis, as demonstrated in Crohn’s disease (17, 18) and psoriasis (19, 148), and if antifungal compounds such as dimethyl fumarate improve the course of spondyloarthritis, as demonstrated in psoriasis (158), psoriatic arthritis (218, 219), and multiple sclerosis (220).

The possible association between *Malassezia* and psoriasis (20, 149, 151, 153, 167, 168) suggests that particular attention should be given to *Malassezia* species in spondyloarthritis. *Malassezia* are ubiquitous facultative intracellular fungi which are difficult to detect. Because of their unique requirement for lipids, they do not grow in commonly used fungal culture media (221). Their DNA is difficult to extract (222), possibly because of their uniquely thick cell wall (221). ITS1 and ITS2, two of the most commonly used primers for fungal consensus PCR, have, respectively, two and one mismatches with *Malassezia* sequences, leading to underrepresentation in PCR products. Recent reports suggest that *Malassezia* are not limited to the skin and are present in the mouth (63), nose (223), gut (71, 138, 170), breast (224), brain (225, 226), and lung (227).

*Malassezia* have the right properties to be able to cause spondyloarthritis: they are ubiquitous, intracellular, present on the skin (65, 166), glans penis (64), mouth (63), and gut (71, 138, 170). *Malassezia* have beta-endorphin receptors which stimulate the secretion of lipases necessary for their growth (228–230), and high beta-endorphin levels are found in the prostate (231–233). *Malassezia* have been strongly associated with granulomatous Crohn’s disease in a pediatric biopsy study (OR = 25.2, 95% CI: 2.45–259, *P* = 0.0025) (138) and with Dectin-1 SNPs in an inflammatory bowel disease study (18). Reads from unidentified Malasseziales were reported in several recent studies of the skin (166, 234) and gut (18), suggesting some species and strains remain to be discovered. To the best of our knowledge, *Malassezia*’s presence in the joints, eyes, and prostate has not been tested.

Because *Malassezia* are common on the human skin, they can be inadvertently inserted in clinical specimens, causing spurious findings. Much care must be taken to ensure that these fungi are actually present in sampled sites. Though our preliminary microbiome results suggest that they are present in the prostate, excluding contamination beyond all doubt has proven to be challenging. As opposed to most infection types, *Malassezia* are ubiquitous and are part of the normal human microbiome, making their association with disease very difficult to prove. For example, distinguishing chronic inflammation caused by autoimmunity against human antigens and immunity against ubiquitous commensal microbe antigens on the psoriatic skin is not trivial, as healthy sites and psoriatic lesions have about the same microbiome (166). Microbes present in more sterile sites such as the joints, eyes, or prostate could provide important clues as to which species may be involved in spondyloarthritis.

Since the evidence available at this time is insufficient to definitely confirm the Catterall–King hypothesis, microbiome studies similar to that performed by Kellermayer et al (138) should be performed to test this hypothesis.

## Author Contributions

First draft was written by ML after discussion with JR and MA. All the authors critiqued and revised the draft.

## Conflict of Interest Statement

The authors declare that the research was conducted in the absence of any commercial or financial relationships that could be construed as a potential conflict of interest.

## References

[1] Schlosstein L Terasaki PI Bluestone R Pearson CM High association of an HL-A antigen, W27, with ankylosing spondylitis. N Engl J Med (1973) 288(14):704–6. 10.1056/NEJM197304052881403 4688372

[2] Brewerton D Hart F Nicholls A Caffrey M James D Sturrock R Ankylosing spondylitis and HL-A 27. Lancet (1973) 301(7809):904–7. 10.1016/S0140-6736(73)91360-3 4123836

[3] Rosenbaum JT Why HLA-B27? My thirty-year quest the Friedenwald lecture. Invest Ophthalmol Vis Sci (2011) 52(10):7712–5. 10.1167/iovs.11-8247 21960642 PMC3183985

[4] Sutcliffe S De Marzo AM Sfanos KS Laurence M. MSMB variation and prostate cancer risk: clues towards a possible fungal etiology. Prostate (2014) 74(6):569–78. 10.1002/pros.22778 24464504 PMC4037912

[5] Benito-Leon J Laurence M The role of fungi in the etiology of multiple sclerosis. Front Neurosci (2017) 8:535 10.3389/fneur.2017.00535 PMC5650687 29085329

[6] Rosenblatt KA Wicklund KG Stanford JL. Sexual factors and the risk of prostate cancer. Am J Epidemiol (2001) 153(12):1152–8. 10.1093/aje/153.12.1152 11415949

[7] Dennis LK Dawson DV. Meta-analysis of measures of sexual activity and prostate cancer. Epidemiology (2002) 13(1):72–9. 10.1097/00001648-200201000-00012 11805589

[8] Taylor ML Mainous AG III Wells BJ. Prostate cancer and sexually transmitted diseases: a meta-analysis. Fam Med (2005) 37(7):506–12. 15988645

[9] Hawkes CH Giovannoni G Keir G Cunnington M Thompson EJ. Seroprevalence of herpes simplex virus type 2 in multiple sclerosis. Acta Neurol Scand (2006) 114(6):363–7. 10.1111/j.1600-0404.2006.00677.x 17083334

[10] Ferrante P Castellani P Barbi M Bergamini F. The Italian Cooperative Multiple Sclerosis case-control study: preliminary results on viral antibodies. Ital J Neurol Sci (1987) 6:45–50. 2820894

[11] Wandinger K Jabs W Siekhaus A Bubel S Trillenberg P Wagner H Association between clinical disease activity and Epstein-Barr virus reactivation in MS. Neurology (2000) 55(2):178–84. 10.1212/WNL.55.2.178 10908887

[12] Catalano LW Herpesvirus hominis antibody in multiple sclerosis and amyotrophic lateral sclerosis. Neurology (1972) 22(5):473–473. 10.1212/WNL.22.5.473 4343501

[13] Strickler HD Goedert JJ Sexual behavior and evidence for an infectious cause of prostate cancer. Epidemiol Rev (2001) 23(1):144–51. 10.1093/oxfordjournals.epirev.a000781 11588840

[14] Hawkes CH. Is multiple sclerosis a sexually transmitted infection? J Neurol Neurosurg Psychiatry (2002) 73(4):439–43. 10.1136/jnnp.73.4.439 12235316 PMC1738067

[15] Stott-Miller M Wright JL Stanford JL. MSMB gene variant alters the association between prostate cancer and number of sexual partners. Prostate (2013) 73(16):1803–9. 10.1002/pros.22719 24037734 PMC3992835

[16] Maillet J Ottaviani S Tubach F Roy C Nicaise-Rolland P Palazzo E Anti-*Saccharomyces cerevisiae* antibodies (ASCA) in spondyloarthritis: prevalence and associated phenotype. Joint Bone Spine (2016) 83(6):665–8. 10.1016/j.jbspin.2015.10.011 26992953

[17] Hoarau G Mukherjee P Gower-Rousseau C Hager C Chandra J Retuerto M Bacteriome and mycobiome interactions underscore microbial dysbiosis in familial Crohn’s disease. mBio (2016) 7(5):e1250–1216. 10.1128/mBio.01250-16 PMC5030358 27651359

[18] Sokol H Leducq V Aschard H Pham H-P Jegou S Landman C Fungal microbiota dysbiosis in IBD. Gut (2016) 66(6):1039–48. 10.1136/gutjnl-2015-310746 26843508 PMC5532459

[19] Waldman A Gilhar A Duek L Berdicevsky I Incidence of *Candida* in psoriasis – a study on the fungal flora of psoriatic patients. Mycoses (2001) 44(3–4):77–81. 10.1046/j.1439-0507.2001.00608.x 11413927

[20] Kanda N Tani K Enomoto U Nakai K Watanabe S. The skin fungus-induced Th1-and Th2-related cytokine, chemokine and prostaglandin E2 production in peripheral blood mononuclear cells from patients with atopic dermatitis and psoriasis vulgaris. Clin Exp Allergy (2002) 32(8):1243–50. 10.1046/j.1365-2745.2002.01459.x 12190666

[21] Taylor-Robinson D Keat A. Observations on *Chlamydia trachomatis* and other microbes in reactive arthritis. Int J STD AIDS (2015) 26(3):139–44. 10.1177/0956462414533319 24828551

[22] Butrimiene I Ranceva J Griskevicius A. Potential triggering infections of reactive arthritis. Scand J Rheumatol (2006) 35(6):459–62. 10.1080/03009740600906750 17343254

[23] Ciurea A Scherer A Weber U Neuenschwander R Tamborrini G Exer P Age at symptom onset in ankylosing spondylitis: is there a gender difference? Ann Rheum Dis (2014) 73(10):1908–10. 10.1136/annrheumdis-2014-205613 25104774

[24] Feldtkeller E Khan MA van der Heijde D van der Linden S Braun J. Age at disease onset and diagnosis delay in HLA-B27 negative vs. positive patients with ankylosing spondylitis. Rheumatol Int (2003) 23(2):61–6. 10.1007/s00296-002-0237-4 12634937

[25] PHE. Sexually Transmitted Infections and Chlamydia Screening in England, 2015. HPR 10(22) Advance Access report (2016). Available from: www.psp94.com/phe_sti_2015.pdf (Accessed: December 7, 2016).

[26] King A Non-gonococcal urethritis and trichomoniasis in the male. Urol Int (1959) 9(3–6):127–45. 10.1159/000277449 14409153

[27] Catterall R Perkins ES Uveitis and urogenital disease in the male. Br J Ophthalmol (1961) 45(2):109 10.1136/bjo.45.2.109 18170649 PMC510043

[28] Catterall R Significance of non-specific genital infection in uveitis and arthritis. Lancet (1961) 278(7205):739–42. 10.1016/S0140-6736(61)90688-2 13877397

[29] Harkness A Reiter’s disease. Br J Vener Dis (1949) 25(4):185. 15398026 10.1136/sti.25.4.185 PMC1053658

[30] Csonka G Reiter’s syndrome. Ergebnisse der inneren Medizin und Kinderheilkunde. Berlin, Heidelberg: Springer (1965). p. 125–89. 10.1007/978-3-642-94912-8_4 5320208

[31] Gillespie CW Manhart LE Lowens MS Golden MR. Asymptomatic urethritis is common and is associated with characteristics that suggest sexually transmitted etiology. Sex Transm Dis (2013) 40(3):271–4. 10.1097/OLQ.0b013e31827c9e42 23407472

[32] Wetmore CM Manhart LE Lowens MS Golden MR Whittington WL Xet-Mull AM Demographic, behavioral, and clinical characteristics of men with nongonococcal urethritis differ by etiology: a case-comparison study. Sex Transm Dis (2011) 38(3):180–6. 10.1097/OLQ.0b013e3182040de9 21285914 PMC4024216

[33] Csonka GW The course of Reiter’s syndrome. Br Med J (1958) 1(5079):1088 10.1136/bmj.1.5079.1088 13536425 PMC2028714

[34] Hall WH Finegold S A study of 23 cases of Reiter’s syndrome. Ann Intern Med (1953) 38(3):533–50. 10.7326/0003-4819-38-3-533 13031398

[35] Ford DK Rasmussen G Relationships between genitourinary infection and complicating arthritis. Arthritis Rheumatol (1964) 7(3):220–7. 10.1002/art.1780070305 14168451

[36] Kvien T Glennås A Melby K Granfors K Andrup O Karstensen B Reactive arthritis: incidence, triggering agents and clinical presentation. J Rheumatol (1994) 21(1):115–22. 8151565

[37] Valtonen V Leirisalo M Pentikäinen P Räsänen T Seppälä I Larinkari U Triggering infections in reactive arthritis. Ann Rheum Dis (1985) 44(6):399–405. 10.1136/ard.44.6.399 3874607 PMC1001660

[38] Söderlin MK Kautiainen H Puolakkainen M Hedman K Söderlund-Venermo M Skogh T Infections preceding early arthritis in southern Sweden: a prospective population-based study. J Rheumatol (2003) 30(3):459–64. 12610801

[39] Csonka G Recurrent attacks in Reiter’s disease. Arthritis Rheumatol (1960) 3(2):164–9. 10.1002/art.1780030208 13813180

[40] Noer HR An experimental epidemic of Reiter’s syndrome. JAMA (1966) 198(7):693–8. 10.1001/jama.198.7.693 5953323

[41] Visher J Chronic prostatitis: its role in etiology of sacroiliac and spinal arthritis. Med J (1929) 130:214–5.

[42] Domeij B Giertz G Olhagen B Romanus R Genitourinary focus in rheumatic disorder in the male. Acta Chir Scand (1958) 115(1–2):1. 13558902

[43] Mason RM Murray RS Oates JK Young AC Prostatitis and ankylosing spondylitis. Br Med J (1958) 1(5073):748–51. 10.1136/bmj.1.5073.748 13510788 PMC2028198

[44] Weinberger H Dienes L Bauer W Clinical features and bacteriological studies in Reiter’s syndrome. Rheumatic Diseases, American Rheumatism Association. Philadelphia, PA: WB Saunders Co. (1952). p. 73–7.

[45] Romanus R Pelvo-spondylitis ossificans in the male and genitourinary infection. Acta Med Scand (1953) 145(S280):178–241. 10.1111/j.0954-6820.1953.tb17256.x

[46] Oates J Incidence of genital infection in male patients with ankylosing spondylitis. Br J Vener Dis (1959) 35(2):89. 14428141 10.1136/sti.35.2.89 PMC1047250

[47] Olhagen B Chronic uro-polyarthritis in the male. Acta Med Scand (1960) 168(4):339–45. 10.1111/j.0954-6820.1960.tb13455.x 13730726

[48] Anonymous. Editorial: ankylosing spondylitis and urogenital infection. Br Med J (1960) 1(5176):865–6. 10.1136/bmj.1.5176.865 13856816 PMC1967081

[49] Catterall R Incidence of chronic genital infection in male patients with uveitis: a preliminary report. Br J Vener Dis (1958) 34(4):254. 13618571 10.1136/sti.34.4.254 PMC1047216

[50] Benedict WL Von Lackum WH Nickel AA The pelvic organs as foci of infection in inflammatory diseases of the eye. Trans Am Ophthalmol Soc (1926) 24:145. 16692727 PMC1316570

[51] Von Lackum WH Clinical and experimental data on prostatic infection. J Urol (1927) 18(3):293–306. 10.1016/S0022-5347(17)73283-1

[52] Zentmayer W The prostate as a remote focus of infection in ocular inflammations. J Am Med Assoc (1926) 87(15):1172–6. 10.1001/jama.1926.02680150006002

[53] Pelouze P Ophthalmologic importance of focal infective prostatitis. Arch Ophthalmol (1932) 7(3):372–7. 10.1001/archopht.1932.00820100026004

[54] Kretschmer H Berkey H Heckel M Ockuly E Chronic prostatitis: a critical review of 1,000 cases. Ill Med J (1937) 71:151–61.

[55] Wright V Reed WB The link between Reiter’s syndrome and psoriatic arthritis. Ann Rheum Dis (1964) 23(1):12 10.1136/ard.23.1.12 14102706 PMC1030844

[56] Perkins E Uveitis in B27-related disease. Ann Rheum Dis (1979) 38(Suppl 1):92 10.1136/ard.38.Suppl_1.92 PMC1049092 318105

[57] Dark A Morton R Acute anterior uveitis in men. Association with chronic prostatitis. Br J Ophthalmol (1968) 52(12):907 10.1136/bjo.52.12.907 5700674 PMC506714

[58] Costantino F Talpin A Said-Nahal R Goldberg M Henny J Chiocchia G Prevalence of spondyloarthritis in reference to HLA-B27 in the French population: results of the GAZEL cohort. Ann Rheum Dis (2015) 74(4):689–93. 10.1136/annrheumdis-2013-204436 24351517

[59] Brown MA Pile KD Kennedy LG Calin A Darke C Bell J HLA class I associations of ankylosing spondylitis in the white population in the United Kingdom. Ann Rheum Dis (1996) 55(4):268–70. 10.1136/ard.55.4.268 8733445 PMC1010149

[60] I. G. o. A. S. Consortium. Identification of multiple risk variants for ankylosing spondylitis through high-density genotyping of immune-related loci. Nat Genet (2013) 45(7):730–8. 10.1038/ng.2667 23749187 PMC3757343

[61] Linssen A Rothova A Valkenburg H Dekker-Saeys A Luyendijk L Kijlstra A The lifetime cumulative incidence of acute anterior uveitis in a normal population and its relation to ankylosing spondylitis and histocompatibility antigen HLA-B27. Invest Ophthalmol Vis Sci (1991) 32(9):2568–78. 1869411

[62] Evans DM Spencer CC Pointon JJ Su Z Harvey D Kochan G Interaction between ERAP1 and HLA-B27 in ankylosing spondylitis implicates peptide handling in the mechanism for HLA-B27 in disease susceptibility. Nat Genet (2011) 43(8):761–7. 10.1038/ng.873 21743469 PMC3640413

[63] Dupuy AK David MS Li L Heider TN Peterson JD Montano EA Redefining the human oral mycobiome with improved practices in amplicon-based taxonomy: discovery of *Malassezia* as a prominent commensal. PLoS One (2014) 9(3):e90899. 10.1371/journal.pone.0090899 24614173 PMC3948697

[64] Mayser P Schütz M Schuppe HC Jung A Schill WB. Frequency and spectrum of *Malassezia* yeasts in the area of the prepuce and glans penis. BJU Int (2001) 88(6):554–8. 10.1046/j.1464-410X.2001.02375.x 11678750

[65] Findley K Oh J Yang J Conlan S Deming C Meyer JA Topographic diversity of fungal and bacterial communities in human skin. Nature (2013) 498:367–70. 10.1038/nature12171 23698366 PMC3711185

[66] Haarr M Rheumatic iridocyclitis. Acta Ophthalmol (1960) 38(1):37–45. 10.1111/j.1755-3768.1960.tb00176.x 13830597

[67] Liang Y Wen H Xiao R Serum levels of antibodies for IgG, IgA, and IgM against the fungi antigen in psoriasis vulgaris. Bull Hunan Med Univ (2003) 28(6):638–40. 15804080

[68] Squiquera L Galimberti R Morelli L Plotkin L Milicich R Kowalckzuk A Antibodies to proteins from *Pityrosporum ovale* in the sera from patients with psoriasis. Clin Exp Dermatol (1994) 19(4):289–93. 10.1111/j.1365-2230.1994.tb01197.x 7955467

[69] Tsoi LC Spain SL Knight J Ellinghaus E Stuart PE Capon F Identification of 15 new psoriasis susceptibility loci highlights the role of innate immunity. Nat Genet (2012) 44(12):1341–8. 10.1038/ng.2467 23143594 PMC3510312

[70] Dotan I Fishman S Dgani Y Schwartz M Karban A Lerner A Antibodies against laminaribioside and chitobioside are novel serologic markers in Crohn’s disease. Gastroenterology (2006) 131(2):366–78. 10.1053/j.gastro.2006.04.030 16890590

[71] Suhr MJ Banjara N Hallen-Adams HE. Sequence-based methods for detecting and evaluating the human gut mycobiome. Lett Appl Microbiol (2016) 62(3):209–15. 10.1111/lam.12539 26669281

[72] Franke A McGovern DP Barrett JC Wang K Radford-Smith GL Ahmad T Genome-wide meta-analysis increases to 71 the number of confirmed Crohn’s disease susceptibility loci. Nat Genet (2010) 42(12):1118–25. 10.1038/ng.717 21102463 PMC3299551

[73] Castro-Santos P Moro-García MA Marcos-Fernández R Alonso-Arias R Díaz-Peña R. ERAP1 and HLA-C interaction in inflammatory bowel disease in the Spanish population. Innate Immun (2017) 23(5):476–81. 10.1177/1753425917716527 28651467

[74] Uhlén M Fagerberg L Hallström BM Lindskog C Oksvold P Mardinoglu A Tissue-based map of the human proteome. Science (2015) 347(6220):1260419 10.1126/science.1260419 25613900

[75] Husby G Tsuchiya N Schwimmbeck PL Keat A Pahle JA Oldstone M Cross-reactive epitope with Klebsiella pneumoniae nitrogenase in articular tissue of HLA–B27+ patients with ankylosing spondylitis. Arthritis Rheumatol (1989) 32(4):437–45. 10.1002/anr.1780320413 2468338

[76] Hannu T. Reactive arthritis. Best Pract Res Clin Rheumatol (2011) 25(3):347–57. 10.1016/j.berh.2011.01.018 22100285

[77] Hughes RA Keat AC Reiter’s syndrome and reactive arthritis: a current view. Semin Arthritis Rheum (1994) 23:190–210. 10.1016/0049-0172(94)90075-2 7534942

[78] Sibilia J Limbach FX. Reactive arthritis or chronic infectious arthritis? Ann Rheum Dis (2002) 61(7):580–7. 10.1136/ard.61.7.580 12079895 PMC1754163

[79] Uotila T Antonen J Laine J Kujansuu E Haapala A Lumio J Reactive arthritis in a population exposed to an extensive waterborne gastroenteritis outbreak after sewage contamination in Pirkanmaa, Finland. Scand J Rheumatol (2011) 40(5):358–62. 10.3109/03009742.2011.562533 21679096

[80] Harkness A Non-gonococcal urethritis. Br J Vener Dis (1933) 9(3):173. 21773522 10.1136/sti.9.3.173 PMC1046792

[81] Thompson SE Washington AE. Epidemiology of sexually transmitted *Chlamydia trachomatis* infections. Epidemiol Rev (1983) 5:96. 10.1093/oxfordjournals.epirev.a036266 6357824

[82] Keat A Thomas BJ Taylor-Robinson D Chlamydial infection in the aetiology of arthritis. Br Med Bull (1983) 39(2):168–74. 10.1093/oxfordjournals.bmb.a071811 6347328

[83] Ostaszewska-Puchalska I Zdrodowska-Stefanow B Kuryliszyn-Moskal A Bułhak-Kozioł V Sokołowska M. Incidence of *Chlamydia trachomatis* infection in patients with reactive arthritis. Reumatologia (2015) 53(2):69. 10.5114/reum.2015.51505 27407230 PMC4847275

[84] Kinsella T Norton W Ziff M Complement-fixing antibodies to bedsonia organisms in Reiter’s syndrome and ankylosing spondylitis. Ann Rheum Dis (1968) 27(3):241 10.1136/ard.27.3.241 5694801 PMC1031101

[85] Ford D Non-gonococcal urethritis and Reiter’s syndrome: personal experience with etiological studies during 15 years. Can Med Assoc J (1968) 99(18):900. 5696921 PMC1945373

[86] Keat A Maini R Nkwazi G Pegrum G Ridgway G Scott J. Role of *Chlamydia trachomatis* and HLA-B27 in sexually acquired reactive arthritis. Br Med J (1978) 1(6113):605–7. 10.1136/bmj.1.6113.605 630254 PMC1603413

[87] Rich E Hook EW Alarcón GS Moreland LW. Reactive arthritis in patients attending an urban sexually transmitted diseases clinic. Arthritis Rheumatol (1996) 39(7):1172–7. 10.1002/art.1780390715 8670327

[88] Van Duynhoven Y Van De Laar M Schop W Mouton J Van der Meijden W Sprenger M. Different demographic and sexual correlates for chlamydial infection and gonorrhoea in Rotterdam. Int J Epidemiol (1997) 26(6):1373–85. 10.1093/ije/26.6.1373 9447420

[89] Satterwhite CL Torrone E Meites E Dunne EF Mahajan R Ocfemia MCB Sexually transmitted infections among US women and men: prevalence and incidence estimates, 2008. Sex Transm Dis (2013) 40(3):187–93. 10.1097/OLQ.0b013e318286bb53 23403598

[90] Clad A Freidank H Kunze M Schnoeckel U Hofmeier S Flecken U Detection of seroconversion and persistence of *Chlamydia trachomatis* antibodies in five different serological tests. Eur J Clin Microbiol Infect Dis (2000) 19(12):932–7. 10.1007/s100960000397 11205630

[91] Rashid T Ebringer A Ankylosing spondylitis is linked to *Klebsiella* – the evidence. Clin Rheumatol (2007) 26(6):858–64. 10.1007/s10067-006-0488-7 17186116

[92] Ebringer R Cawdell D Cowling P Ebringer A. Sequential studies in ankylosing spondylitis. Association of *Klebsiella pneumoniae* with active disease. Ann Rheum Dis (1978) 37(2):146–51. 10.1136/ard.37.2.146 348130 PMC1001180

[93] White L McCoy R Tait B Ebringer R. A search for gram-negative enteric micro-organisms in acute anterior uveitis: association of klebsiella with recent onset of disease, HLA-B27, and B7 CREG. Br J Ophthalmol (1984) 68(10):750–5. 10.1136/bjo.68.10.750 6332644 PMC1040459

[94] Smith G Blackwell C Nuki G. Faecal flora in spondyloarthropathy. Br J Rheumatol (1997) 36(8):850–4. 10.1093/rheumatology/36.8.850 9291853

[95] Park H Schumacher H Zeiger A Rosenbaum J. Antibodies to peptidoglycan in patients with spondylarthritis: a clue to disease aetiology? Ann Rheum Dis (1984) 43(5):725–8. 10.1136/ard.43.5.725 6497464 PMC1001516

[96] Rahman M Ahmed S Schumacher H Zeiger A. High levels of antipeptidoglycan antibodies in psoriatic and other seronegative arthritides. J Rheumatol (1990) 17(5):621–5. 2359072

[97] Schiefer HG Weidner W Krauss H Gerhardt U Schmidt KL. Rheumatoid factor-negative arthritis, especially ankylosing spondylitis, and infections of the male urogenital tract. Zentralbl Bakteriol Mikrobiol Hyg A (1983) 255(4):511–7. 6659736

[98] Van der Paardt M van Denderen J van den Brule A Morré S van der Horst-Bruinsma I Bezemer P Prevalence of *Chlamydia trachomatis* in urine of male patients with ankylosing spondylitis is not increased. Ann Rheum Dis (2000) 59(4):300–2. 10.1136/ard.59.4.300 10733479 PMC1753118

[99] Lange U Berliner M Ludwig M Schiefer H Teichmann J Weidner W Ankylosing spondylitis and infections of the female urogenital tract. Rheumatol Int (1998) 17(5):181–4. 10.1007/s002960050031 9542778

[100] Csango P Upsahl M Romberg Ø Kornstad L Sarov I. *Chlamydia trachomatis* serology in ankylosing spondylitis. Clin Rheumatol (1987) 6(3):384–90. 10.1007/BF02206837 3327642

[101] Savolainen E Kettunen A Närvänen A Kautiainen H Kärkkäinen U Luosujärvi R Prevalence of antibodies against *Chlamydia trachomatis* and incidence of *C. trachomatis*-induced reactive arthritis in an early arthritis series in Finland in 2000. Scand J Rheumatol (2009) 38(5):353–6. 10.1080/03009740902769559 19296404

[102] Hoffman I Demetter P Peeters M De Vos M Mielants H Veys EM Anti-*Saccharomyces cerevisiae* IgA antibodies are raised in ankylosing spondylitis and undifferentiated spondyloarthropathy. Ann Rheum Dis (2003) 62(5):455–9. 10.1136/ard.62.5.455 12695160 PMC1754530

[103] Török H-P Glas J Gruber R Brumberger V Strasser C Kellner H Inflammatory bowel disease-specific autoantibodies in HLA-B27-associated spondyloarthropathies: increased prevalence of ASCA and pANCA. Digestion (2004) 70(1):49–54. 10.1159/000080081 15308872

[104] Aydin S Atagunduz P Temel M Bicakcigil M Tasan D Direskeneli H. Anti-*Saccharomyces cerevisiae* antibodies (ASCA) in spondyloarthropathies: a reassessment. Rheumatology (2008) 47(2):142–4. 10.1093/rheumatology/kem324 18160421

[105] Mundwiler ML Mei L Landers CJ Reveille JD Targan S Weisman MH. Inflammatory bowel disease serologies in ankylosing spondylitis patients: a pilot study. Arthritis Res Ther (2009) 11(6):R177. 10.1186/ar2866 19930665 PMC3003540

[106] Riente L Chimenti D Pratesi F Delle Sedie A Tommasi S Tommasi C Antibodies to tissue transglutaminase and *Saccharomyces cerevisiae* in ankylosing spondylitis and psoriatic arthritis. J Rheumatol (2004) 31(5):920–4. 15124251

[107] Huhtinen M Laasila K Granfors K Puolakkainen M Seppälä I Laasonen L Infectious background of patients with a history of acute anterior uveitis. Ann Rheum Dis (2002) 61(11):1012–6. 10.1136/ard.61.11.1012 12379526 PMC1753942

[108] Onal S Kazokoglu H Incili B Demiralp EE Yavuz S. Prevalence and levels of serum antibodies to gram negative microorganisms in Turkish patients with HLA-B27 positive acute anterior uveitis and controls. Ocul Immunol Inflamm (2006) 14(5):293–9. 10.1080/09273940600977241 17056463

[109] Brown MA Kennedy LG MacGregor AJ Darke C Duncan E Shatford JL Susceptibility to ankylosing spondylitis in twins: the role of genes, HLA, and the environment. Arthritis Rheum (1997) 40(10):1823–8. 10.1002/art.1780401015 9336417

[110] Ruutu M Thomas G Steck R Degli-Esposti MA Zinkernagel MS Alexander K β-glucan triggers spondylarthritis and Crohn’s disease–like ileitis in SKG mice. Arthritis Rheum (2012) 64(7):2211–22. 10.1002/art.34423 22328069

[111] Chesson HW Dunne EF Hariri S Markowitz LE. The estimated lifetime probability of acquiring human papillomavirus in the United States. Sex Transm Dis (2014) 41(11):660–4. 10.1097/OLQ.0000000000000193 25299412 PMC6745688

[112] Van Metre TE Brown WH Knox DL Maumenee AE The relation between nongranulomatous uveitis and arthritis. J Allergy (1965) 36(2):158–74. 10.1016/0021-8707(65)90164-4 14269552

[113] Huhtinen M Karma A. HLA-B27 typing in the categorisation of uveitis in a HLA-B27 rich population. Br J Ophthalmol (2000) 84(4):413–6. 10.1136/bjo.84.4.413 10729301 PMC1723428

[114] Popert A Gill A Laird S A prospective study of Reiter’s syndrome: an interim report on the first 82 cases. Br J Vener Dis (1964) 40(3):160. 14210482 10.1136/sti.40.3.160 PMC1047645

[115] Eastmond C. Gram-negative bacteria and B27 disease. Rheumatology (1983) 22(Suppl_2):67–74. 10.1093/rheumatology/XXII.suppl_2.67 6606475

[116] Hannu T Mattila L Siitonen A Leirisalo-Repo M. Reactive arthritis attributable to *Shigella* infection: a clinical and epidemiological nationwide study. Ann Rheum Dis (2005) 64(4):594–8. 10.1136/ard.2004.027524 15550534 PMC1755450

[117] Rudwaleit M Richter S Braun J Sieper J. Low incidence of reactive arthritis in children following a salmonella outbreak. Ann Rheum Dis (2001) 60(11):1055–7. 10.1136/ard.60.11.1055 11602478 PMC1753416

[118] Smeekens SP Plantinga TS van de Veerdonk FL Heinhuis B Hoischen A Joosten LA STAT1 hyperphosphorylation and defective IL12R/IL23R signaling underlie defective immunity in autosomal dominant chronic mucocutaneous candidiasis. PLoS One (2011) 6(12):e29248. 10.1371/journal.pone.0029248 22195034 PMC3237610

[119] Puel A Cypowyj S Bustamante J Wright JF Liu L Lim HK Chronic mucocutaneous candidiasis in humans with inborn errors of interleukin-17 immunity. Science (2011) 332(6025):65–8. 10.1126/science.1200439 21350122 PMC3070042

[120] Glocker E-O Hennigs A Nabavi M Schäffer AA Woellner C Salzer U A homozygous CARD9 mutation in a family with susceptibility to fungal infections. N Engl J Med (2009) 361(18):1727–35. 10.1056/NEJMoa0810719 19864672 PMC2793117

[121] Saunte D Mrowietz U Puig L Zachariae C. *Candida* infections in patients with psoriasis and psoriatic arthritis treated with interleukin-17 inhibitors and their practical management. Br J Dermatol (2017) 177(1):47–62. 10.1111/bjd.15015 27580411

[122] Romani L Immunity to fungal infections. Nat Rev Immunol (2011) 11(4):275–88. 10.1038/nri2939 21394104

[123] Brown BR Lee EJ Snow PE Vance EE Iwakura Y Ohno N Fungal-derived cues promote ocular autoimmunity through a Dectin-2/Card9-mediated mechanism. Clin Exp Immunol (2017) 190(3):293–303. 10.1111/cei.13021 28763100 PMC5680064

[124] Suzuki T Ohno N Ohshima Y Yadomae T Soluble mannan and β-glucan inhibit the uptake of *Malassezia furfur* by human monocytic cell line, THP-1. FEMS Immunol Med Microbiol (1998) 21(3):223–30. 10.1111/j.1574-695X.1998.tb01169.x 9718212

[125] Cross C Bancroft G. Ingestion of acapsular *Cryptococcus neoformans* occurs via mannose and beta-glucan receptors, resulting in cytokine production and increased phagocytosis of the encapsulated form. Infect Immun (1995) 63(7):2604–11. 7790075 10.1128/iai.63.7.2604-2611.1995 PMC173349

[126] Baeten D Baraliakos X Braun J Sieper J Emery P Van Der Heijde D Anti-interleukin-17A monoclonal antibody secukinumab in treatment of ankylosing spondylitis: a randomised, double-blind, placebo-controlled trial. Lancet (2013) 382(9906):1705–13 10.1016/S0140-6736(13)61134-4 24035250

[127] Cozon GJN Mbitikon-Kobo FM Fatoohi F Spire M Grange J-D Kodjikian L. Abnormal cellular reactivity to microbial antigens in patients with uveitis. Invest Ophthalmol Vis Sci (2008) 49(6):2526–30. 10.1167/iovs.07-1454 18362113

[128] Messenger W Hildebrandt L Mackensen F Suhler E Becker M Rosenbaum JT.Characterisation of uveitis in association with multiple sclerosis. Br J Ophthalmol (2015) 99(2):205–9. 10.1136/bjophthalmol-2014-305518 25170065

[129] Tang WM Pulido JS Eckels DD Han DP Mieler WF Pierce K. The association of HLA-DR15 and intermediate uveitis. Am J Ophthalmol (1997) 123(1):70–5. 10.1016/S0002-9394(14)70994-8 9186099

[130] Heinz C Heiligenhaus A Improvement of noninfectious uveitis with fumaric acid esters: results of a pilot study. Arch Ophthalmol (2007) 125(4):569–71. 10.1001/archopht.125.4.569 17420383

[131] Barisani-Asenbauer T Maca SM Mejdoubi L Emminger W Machold K Auer H. Uveitis-a rare disease often associated with systemic diseases and infections-a systematic review of 2619 patients. Orphanet J Rare Dis (2012) 7(1):57. 10.1186/1750-1172-7-57 22932001 PMC3503654

[132] Cosnes J Gower–Rousseau C Seksik P Cortot A. Epidemiology and natural history of inflammatory bowel diseases. Gastroenterology (2011) 140(6):1785–1794. 10.1053/j.gastro.2011.01.055 21530745

[133] Van Praet L Van den Bosch FE Jacques P Carron P Jans L Colman R Microscopic gut inflammation in axial spondyloarthritis: a multiparametric predictive model. Ann Rheum Dis (2013) 72(3):414–7. 10.1136/annrheumdis-2012-202135 23139267

[134] Jyonouchi H Geng L Cushing-Ruby A Monteiro IM. Aberrant responses to TLR agonists in pediatric IBD patients; the possible association with increased production of Th1/Th17 cytokines in response to candida, a luminal antigen. Pediatr Allergy Immunol (2010) 21(4p2):e747–55. 10.1111/j.1399-3038.2009.00923.x 19725895

[135] Baram L Cohen-Kedar S Spektor L Elad H Guzner-Gur H Dotan I. Differential stimulation of peripheral blood mononuclear cells in Crohn’s disease by fungal glycans. J Gastroenterol Hepatol (2014) 29(12):1976–84. 10.1111/jgh.12701 25092526

[136] Hegazy AN West NR Stubbington MJT Wendt E Suijker KIM Datsi A Circulating and tissue-resident CD4+ T cells with reactivity to intestinal microbiota are abundant in healthy individuals and function is altered during inflammation. Gastroenterology (2017) 153(5):1320–37.e16. 10.1053/j.gastro.2017.07.047 28782508 PMC5687320

[137] Gerard R Sendid B Colombel J-F Poulain D Jouault T An immunological link between *Candida albicans* colonization and Crohn’s disease. Crit Rev Microbiol (2015) 41(2):135–9. 10.3109/1040841X.2013.810587 23855357

[138] Kellermayer R Mir SA Nagy-Szakal D Cox SB Dowd SE Kaplan JL Microbiota separation and C-reactive protein elevation in treatment naïve pediatric granulomatous Crohn disease. J Pediatric Gastroenterol Nutr (2012) 55(3):243–50. 10.1097/MPG.0b013e3182617c16 PMC3812911 22699834

[139] Varkas G Thevissen K De Brabanter G Van Praet L Czul-Gurdian F Cypers H An induction or flare of arthritis and/or sacroiliitis by vedolizumab in inflammatory bowel disease: a case series. Ann Rheum Dis (2017) 76(5):878–81. 10.1136/annrheumdis-2016-210233 27899374

[140] Wendling D Sondag M Verhoeven F Vuitton L Koch S Prati C Arthritis occurrence or reactivation under Vedolizumab treatment for inflammatory bowel disease. A four cases report. Joint Bone Spine (2017) 85(2):255–6. 10.1016/j.jbspin.2017.01.012 28238881

[141] Tadbiri S Peyrin-Biroulet L Serrero M Filippi J Pariente B Roblin X Impact of vedolizumab therapy on extra-intestinal manifestations in patients with inflammatory bowel disease: a multicentre cohort study nested in the OBSERV-IBD cohort. Aliment Pharmacol Ther (2017) 47(4):485–93. 10.1111/apt.14419 29250803

[142] Salmi M Jalkanen S. Human leukocyte subpopulations from inflamed gut bind to joint vasculature using distinct sets of adhesion molecules. J Immunol (2001) 166(7):4650–7. 10.4049/jimmunol.166.7.4650 11254724

[143] Ciccia F Guggino G Rizzo A Saieva L Peralta S Giardina A Type 3 innate lymphoid cells producing IL-17 and IL-22 are expanded in the gut, in the peripheral blood, synovial fluid and bone marrow of patients with ankylosing spondylitis. Ann Rheum Dis (2015) 74(9):1739–47. 10.1136/annrheumdis-2014-206323 25902790

[144] Salmi M Jalkanen S. Endothelial ligands and homing of mucosal leukocytes in extraintestinal manifestations of IBD. Inflamm Bowel Dis (1998) 4(2):149–56. 10.1097/00054725-199805000-00026 9589300

[145] Chouraki V Savoye G Dauchet L Vernier-Massouille G Dupas J-L Merle V The changing pattern of Crohn’s disease incidence in northern France: a continuing increase in the 10- to 19-year-old age bracket (1988–2007). Aliment Pharmacol Ther (2011) 33(10):1133–42. 10.1111/j.1365-2036.2011.04628.x 21488915

[146] Yao T Matsui T Hiwatashi N Crohn’s disease in Japan. Dis Colon Rectum (2000) 43:S85–93. 10.1007/BF02237231 11052483

[147] Parisi R Symmons DP Griffiths CE Ashcroft DM. Global epidemiology of psoriasis: a systematic review of incidence and prevalence. J Invest Dermatol (2013) 133(2):377–85. 10.1038/jid.2012.339 23014338

[148] Sepahi S Riahi-Zanjani B Ghorani A The role of *Candida albicans* in the pathogenesis of *Psoriasis vulgaris*: systematic literature review. Rev Clin Med (2016) 3:122–7. 10.22038/rcm.2016.6485

[149] Lober CW Belew PW Rosenberg EW Bale G. Patch tests with killed sonicated microflora in patients with psoriasis. Arch Dermatol (1982) 118(5):322–5. 10.1001/archderm.1982.01650170036019 6211147

[150] Imai Y Tsuda T Aochi S Futatsugi-Yumikura S Sakaguchi Y Nakagawa N YKL-40 (chitinase 3-like-1) as a biomarker for psoriasis vulgaris and pustular psoriasis. J Dermatol Sci (2011) 64(1):75–7. 10.1016/j.jdermsci.2011.06.012 21782391

[151] Faergemann J Treatment of Sebopsoriasis with Itraconazole: Behandlung von Sebopsoriasis mit Itraconazole. Mycoses (1985) 28(12):612–8. 10.1111/j.1439-0507.1985.tb02094.x 3003569

[152] Rosenberg EW Belew PW Improvement of psoriasis of the scalp with ketoconazole. Arch Dermatol (1982) 118(6):370–1. 10.1001/archderm.118.6.370 6284060

[153] Farr P Marks J Krause L Shuster S. Response of scalp psoriasis to oral ketoconazole. Lancet (1985) 326(8461):921–2. 10.1016/S0140-6736(85)90853-0 2865422

[154] Crutcher N Rosenberg EW Belew PW Skinner RB Jr Eaglstein NF Baker SM Oral nystatin in the treatment of psoriasis. Arch Dermatol (1984) 120(4):435–6. 10.1001/archderm.120.4.435a 6703746

[155] Ganor S Treatment of early psoriasis lesions with a oral amphotericin B or nystatin. Int J Dermatol (1988) 27(6):420–420. 10.1111/j.1365-4362.1988.tb02398.x

[156] Buslau M Hänel H Holzmann H The significance of yeasts in seborrheic eczema. Hautarzt (1989) 40(10):611–3. 2533189

[157] Ascioglu O Soyuer U Aktas E Improvement of psoriasis with oral nystatin. In: Tümbay E Seeliger HPR Anǧ Ö, editors. Candida and Candidamycosis. Boston, MA: Springer (1991). p. 279–81. 10.1007/978-1-4684-5910-4_48

[158] Balak D Fallah Arani S Hajdarbegovic E Hagemans C Bramer W Thio H Efficacy, effectiveness and safety of fumaric acid esters in the treatment of psoriasis: a systematic review of randomized and observational studies. Br J Dermatol (2016) 175(2):250–62. 10.1111/bjd.14500 26919824

[159] Chiu H-Y Chang W-L Tsai T-F Tsai Y-W Shiu M-N. Risk of psoriasis following terbinafine or itraconazole treatment for onychomycosis: a population-based case-control comparative study. Drug Safety (2018) 41(3):285–95. 10.1007/s40264-017-0614-2 29110252

[160] Swanbeck G Inerot A Martinsson T Wahlström J Enerbäck C Enlund F Age at onset and different types of psoriasis. Br J Dermatol (1995) 133(5):768–73. 10.1111/j.1365-2133.1995.tb02753.x 8555031

[161] Rahman P Schentag CT Gladman DD Immunogenetic profile of patients with psoriatic arthritis varies according to the age at onset of psoriasis. Arthritis Rheumatol (1999) 42(4):818–23. 10.1002/1529-0131(199904)42:4<818::AID-ANR30>3.0.CO;2-5 10211902

[162] Lysell J Padyukov L Kockum I Nikamo P Ståhle M. Genetic association with ERAP1 in psoriasis is confined to disease onset after puberty and not dependent on HLA-C* 06. J Invest Dermatol (2013) 133(2):411–7. 10.1038/jid.2012.280 22931917 PMC3547223

[163] Adachi A Horikawa T Ichihashi M Takashima T Komura A Role of *Candida allergen* in atopic dermatitis and efficacy of oral therapy with various antifungal agents. Allergy (1999) 48(7):719–25. 10481356

[164] Larson JL Wallace TL Tyl RW Marr MC Myers CB Cossum PA The reproductive and developmental toxicity of the antifungal drug Nyotran^®^ (liposomal nystatin) in rats and rabbits. Toxicol Sci (2000) 53(2):421–9. 10.1093/toxsci/53.2.421 10696790

[165] Pauls K Schön M Kubitza RC Homey B Wiesenborn A Lehmann P Role of integrin α E (CD103) β 7 for tissue-specific epidermal localization of CD8+ T lymphocytes. J Invest Dermatol (2001) 117(3):569–75. 10.1046/j.0022-202x.2001.01481.x 11564161

[166] Tett A Pasolli E Farina S Truong DT Asnicar F Zolfo M Unexplored diversity and strain-level structure of the skin microbiome associated with psoriasis. NPJ Biofilms Microbiomes (2017) 3:1–12. 10.1038/s41522-017-0022-5 28649415 PMC5481418

[167] Gomez-Moyano E Crespo-Erchiga V Martínez-Pilar L Diaz DG Martínez-García S Navarro ML Do *Malassezia* species play a role in exacerbation of scalp psoriasis? J Mycol Med (2014) 24(2):87–92. 10.1016/j.mycmed.2013.10.007 24411177

[168] Fry L Baker BS. Triggering psoriasis: the role of infections and medications. Clin Dermatol (2007) 25(6):606–15. 10.1016/j.clindermatol.2007.08.015 18021899

[169] Pierard J Dockx P The ultrastructure of tinea versicolor and *Malassezia furfur*. Int J Dermatol (1972) 11(2):116–24. 10.1111/j.1365-4362.1972.tb01735.x 4554182

[170] Hallen-Adams HE Kachman SD Kim J Legge RM Martínez I Fungi inhabiting the healthy human gastrointestinal tract: a diverse and dynamic community. Fungal Ecol (2015) 15:9–17. 10.1016/j.funeco.2015.01.006

[171] de la Rosette JJ Hubregtse MR Meuleman EJ Stolk-Engelaar MV Debruyne FM. Diagnosis and treatment of 409 patients with prostatitis syndromes. Urology (1993) 41(4):301–7. 10.1016/0090-4295(93)90584-W 8470312

[172] Edstrom Hagerwall AM Rydengard V Fernlund P Morgelin M Baumgarten M Cole AM Beta-microseminoprotein endows post coital seminal plasma with potent candidacidal activity by a calcium- and pH-dependent mechanism. PLoS Pathog (2012) 8(4):e1002625 10.1371/journal.ppat.1002625 22496651 PMC3320615

[173] Eeles RA Kote-Jarai Z Giles GG Olama AA Guy M Jugurnauth SK Multiple newly identified loci associated with prostate cancer susceptibility. Nat Genet (2008) 40(3):316–21. 10.1038/ng.90 18264097

[174] Kote-Jarai Z Easton DF Stanford JL Ostrander EA Schleutker J Ingles SA Multiple novel prostate cancer predisposition loci confirmed by an international study: the PRACTICAL Consortium. Cancer Epidemiol Biomarkers Prev (2008) 17(8):2052–61. 10.1158/1055-9965.EPI-08-0317 18708398 PMC2776652

[175] Thomas G Jacobs KB Yeager M Kraft P Wacholder S Orr N Multiple loci identified in a genome-wide association study of prostate cancer. Nat Genet (2008) 40(3):310–5. 10.1038/ng.91 18264096

[176] Xu X Valtonen-Andre C Savblom C Hallden C Lilja H Klein RJ. Polymorphisms at the microseminoprotein-beta locus associated with physiologic variation in beta-microseminoprotein and prostate-specific antigen levels. Cancer Epidemiol Biomarkers Prev (2010) 19(8):2035–42. 10.1158/1055-9965.EPI-10-0431 20696662 PMC2946372

[177] Hrbacek J Urban M Hamsikova E Tachezy R Heracek J Thirty years of research on infection and prostate cancer: no conclusive evidence for a link. A systematic review. Urol Oncol (2012) 31(7):951–65. 10.1016/j.urolonc.2012.01.013 22459691

[178] Xu K Wang X Ling MT Lee DT Fan T Chan FL Identification of a specifically expressed modified form of novel PSP-94 protein in the secretion of benign prostatic hyperplasia. Electrophoresis (2003) 24(7–8):1311–8. 10.1002/elps.200390167 12707925

[179] Tsai Y Harrison H Lee C Daufeldt J Oliver L Grayhack J. Systematic characterization of human prostatic fluid proteins with two-dimensional electrophoresis. Clin Chem (1984) 30(12):2026–30. 6209034

[180] Doctor VM Sheth AR Simha MM Arbatti NJ Aaveri JP Sheth NA. Studies on immunocytochemical localization of inhibin-like material in human prostatic tissue: comparison of its distribution in normal, benign and malignant prostates. Br J Cancer (1986) 53(4):547–54. 10.1038/bjc.1986.85 2423106 PMC2001445

[181] Sathe VS Sheth NA Phadke MA Sheth AR Zaveri JP. Biosynthesis and localization of inhibin in human prostate. Prostate (1987) 10(1):33–43. 10.1002/pros.2990100107 2434935

[182] Kramer G Marberger M. Could inflammation be a key component in the progression of benign prostatic hyperplasia? Curr Opin Urol (2006) 16(1):25–9. 10.1097/01.mou.0000193368.91823.1b 16385197

[183] Guess HA Arrighi HM Metter EJ Fozard JL. Cumulative prevalence of prostatism matches the autopsy prevalence of benign prostatic hyperplasia. Prostate (1990) 17(3):241–6. 10.1002/pros.2990170308 1700403

[184] Seman G Gallager HS Johnson DE. Melanin-like pigment in the human prostate. Prostate (1982) 3(1):59–72. 10.1002/pros.2990030109 6176988

[185] Guillan R Zelman S The incidence and probable origin of melanin in the prostate. J Urol (1970) 104(1):151–3. 10.1016/S0022-5347(17)61689-6 4193408

[186] Maksem JA Johenning PW Galang CF Prostatitis and aspiration biopsy cytology of prostate. Urology (1988) 32(3):263–8. 10.1016/0090-4295(88)90398-6 3413918

[187] Gómez BL Nosanchuk JD Melanin and fungi. Curr Opin Infect Dis (2003) 16(2):91–6. 10.1097/00001432-200304000-00005 12734441

[188] Fernandes C Prados-Rosales R Silva BM Nakouzi-Naranjo A Zuzarte M Chatterjee S Activation of melanin synthesis in Alternaria infectoria by antifungal drugs. Antimicrob Agents Chemother (2016) 60(3):1646–55. 10.1128/AAC.02190-15 PMC4775914 26711773

[189] Weiber H Andersson C Murne A Rannevik G Lindstrom C Lilja H Beta microseminoprotein is not a prostate-specific protein. Its identification in mucous glands and secretions. Am J Pathol (1990) 137(3):593–603. 2205099 PMC1877516

[190] Ohkubo I Tada T Ochiai Y Ueyama H Eimoto T Sasaki M. Human seminal plasma beta-microseminoprotein: its purification, characterization, and immunohistochemical localization. Int J Biochem Cell Biol (1995) 27(6):603–11. 10.1016/1357-2725(95)00021-G 7671139

[191] Nolet S St-Louis D Mbikay M Chretien M. Rapid evolution of prostatic protein PSP94 suggested by sequence divergence between rhesus monkey and human cDNAs. Genomics (1991) 9(4):775–7. 10.1016/0888-7543(91)90375-O 2037304

[192] Ghasriani H Teilum K Johnsson Y Fernlund P Drakenberg T. Solution structures of human and porcine beta-microseminoprotein. J Mol Biol (2006) 362(3):502–15. 10.1016/j.jmb.2006.07.029 16930619

[193] Clark NL Swanson WJ. Pervasive adaptive evolution in primate seminal proteins. PLoS Genet (2005) 1(3):e35. 10.1371/journal.pgen.0010035 16170411 PMC1201370

[194] Pearson CM Development of arthritis, periarthritis and periostitis in rats given adjuvants. Proc Soc Exp Biol Med (1956) 91(1):95–101. 10.3181/00379727-91-22179 13297719

[195] Pearson CM Wood FD Studies of polyarthritis and other lesions induced in rats by injection of mycobacterial adjuvant. I. General clinical and pathologic characteristics and some modifying factors. Arthritis Rheumatol (1959) 2(5):440–59. 10.1002/1529-0131(195910)2:5<440::AID-ART1780020510>3.0.CO;2-N

[196] Taurog JD Sandberg GP Mahowald ML. The cellular basis of adjuvant arthritis: II. Characterization of the cells mediating passive transfer. Cell Immunol (1983) 80(1):198–204. 10.1016/0008-8749(83)90106-5 6603276

[197] Waksman BH Pearson CM Sharp JT Studies of arthritis and other lesions induced in rats by injection of mycobacterial adjuvant. J Immunol (1960) 85(4):403–17. 13782608

[198] Taurog JD Rival C van Duivenvoorde LM Satumtira N Dorris ML Sun M Autoimmune epididymoorchitis is essential to the pathogenesis of male-specific spondylarthritis in HLA–B27–transgenic rats. Arthritis Rheumatol (2012) 64(8):2518–28. 10.1002/art.34480 PMC3396784 22488218

[199] Sonoyama K Miki A Sugita R Goto H Nakata M Yamaguchi N. Gut colonization by *Candida albicans* aggravates inflammation in the gut and extra-gut tissues in mice. Med Mycol (2011) 49(3):237–47. 10.3109/13693786.2010.511284 20807027

[200] Hida S Miura NN Adachi Y Ohno N Cell wall β-glucan derived from *Candida albicans* acts as a trigger for autoimmune arthritis in SKG mice. Biol Pharm Bull (2007) 30(8):1589–92. 10.1248/bpb.30.1589 17666828

[201] Marijnissen RJ Koenders MI Van de Veerdonk FL Dulos J Netea MG Boots AM Exposure to *Candida albicans* polarizes a T-cell driven arthritis model towards Th17 responses, resulting in a more destructive arthritis. PLoS One (2012) 7(6):e38889. 10.1371/journal.pone.0038889 22719976 PMC3373564

[202] Miyake Y Toyonaga K Mori D Kakuta S Hoshino Y Oyamada A C-type lectin MCL is an FcRγ-coupled receptor that mediates the adjuvanticity of mycobacterial cord factor. Immunity (2013) 38(5):1050–62. 10.1016/j.immuni.2013.03.010 23602766

[203] Lee EJ Brown BR Vance EE Snow PE Silver PB Heinrichs D Mincle activation and the Syk/Card9 signaling axis are central to the development of autoimmune disease of the eye. J Immunol (2016) 196(7):3148–58. 10.4049/jimmunol.1502355 26921309 PMC4799727

[204] Yamasaki S Matsumoto M Takeuchi O Matsuzawa T Ishikawa E Sakuma M C-type lectin Mincle is an activating receptor for pathogenic fungus, Malassezia. Proc Natl Acad Sci U S A (2009) 106(6):1897–902. 10.1073/pnas.0805177106 19171887 PMC2644135

[205] Lord JC Hartzer KL Kambhampati S. A nuptially transmitted ichthyosporean symbiont of *Tenebrio molitor* (Coleoptera: Tenebrionidae). J Eukaryot Microbiol (2012) 59(3):246–50. 10.1111/j.1550-7408.2012.00617.x 22510059

[206] Ramos M Pisa D Molina S Rábano A Juarranz Á Carrasco L Fungal infection in patients with multiple sclerosis. Open Mycol J (2008) 2(1):22–8. 10.2174/1874437000802010022

[207] Benito-Leon J Pisa D Alonso R Calleja P Diaz-Sanchez M Carrasco L. Association between multiple sclerosis and *Candida* species: evidence from a case-control study. Eur J Clin Microbiol Infect Dis (2010) 29(9):1139–45. 10.1007/s10096-010-0979-y 20556470

[208] Pisa D Alonso R Jimenez-Jimenez FJ Carrasco L. Fungal infection in cerebrospinal fluid from some patients with multiple sclerosis. Eur J Clin Microbiol Infect Dis (2013) 32(6):795–801. 10.1007/s10096-012-1810-8 23322279

[209] Guerini FR Cagliani R Forni D Agliardi C Caputo D Cassinotti A A functional variant in ERAP1 predisposes to multiple sclerosis. PLoS One (2012) 7(1):e29931. 10.1371/journal.pone.0029931 22253828 PMC3257233

[210] Babbe H Roers A Waisman A Lassmann H Goebels N Hohlfeld R Clonal expansions of CD8+ T cells dominate the T cell infiltrate in active multiple sclerosis lesions as shown by micromanipulation and single cell polymerase chain reaction. J Exp Med (2000) 192(3):393–404. 10.1084/jem.192.3.393 10934227 PMC2193223

[211] Hook EW III Marra CM Acquired syphilis in adults. N Engl J Med (1992) 326(16):1060–9. 10.1056/NEJM199204163261606 1549153

[212] Palacios G Druce J Du L Tran T Birch C Briese T A new arenavirus in a cluster of fatal transplant-associated diseases. N Engl J Med (2008) 358(10):991–8. 10.1056/NEJMoa073785 18256387

[213] Lipkin WI. Microbe hunting. Microbiol Mol Biol Rev (2010) 74(3):363–77. 10.1128/MMBR.00007-10 20805403 PMC2937520

[214] Cottier F Srinivasan KG Yurieva M Liao W Poidinger M Zolezzi F Advantages of meta-total RNA sequencing (MeTRS) over shotgun metagenomics and amplicon-based sequencing in the profiling of complex microbial communities. NPJ Biofilms Microbiomes (2018) 4(1):2. 10.1038/s41522-017-0046-x 29367879 PMC5773663

[215] Kowarsky M Camunas-Soler J Kertesz M De Vlaminck I Koh W Pan W Numerous uncharacterized and highly divergent microbes which colonize humans are revealed by circulating cell-free DNA. Proc Natl Acad Sci U S A (2017) 114(36):9623–8. 10.1073/pnas.1707009114 28830999 PMC5594678

[216] Ghannoum MA Jurevic RJ Mukherjee PK Cui F Sikaroodi M Naqvi A Characterization of the oral fungal microbiome (mycobiome) in healthy individuals. PLoS Pathog (2010) 6(1):e1000713. 10.1371/journal.ppat.1000713 20072605 PMC2795202

[217] Guo R Zheng N Lu H Yin H Yao J Chen Y. Increased diversity of fungal flora in the vagina of patients with recurrent vaginal candidiasis and allergic rhinitis. Microb Ecol (2012) 64(4):918–27. 10.1007/s00248-012-0084-0 22767123

[218] Peeters A Dukmans B Van der Schroeff J Fumaric acid therapy for psoriatic arthritis. A randomized, double-blind, placebo-controlled study. Rheumatology (1992) 31(7):502–4. 10.1093/rheumatology/31.7.502 1628175

[219] Altmeyer PJ Mattlies U Pawlak F Hoffmann K Frosch PJ Ruppert P Antipsoriatic effect of fumaric acid derivatives: results of a multicenter double-blind study in 100 patients. J Am Acad Dermatol (1994) 30(6):977–81. 10.1016/S0190-9622(94)70121-0 8188891

[220] Gold R Kappos L Arnold DL Bar-Or A Giovannoni G Selmaj K Placebo-controlled phase 3 study of oral BG-12 for relapsing multiple sclerosis. N Engl J Med (2012) 367(12):1098–107. 10.1056/NEJMoa1114287 22992073

[221] Ashbee HR Evans EGV. Immunology of diseases associated with *Malassezia* species. Clin Microbiol Rev (2002) 15(1):21–57. 10.1128/CMR.15.1.21-57.2002 11781265 PMC118058

[222] Vesty A Biswas K Taylor MW Gear K Douglas RG. Evaluating the impact of DNA extraction method on the representation of human oral bacterial and fungal communities. PLoS One (2017) 12(1):e0169877. 10.1371/journal.pone.0169877 28099455 PMC5242530

[223] Cleland EJ Bassioni A Boase S Dowd S Vreugde S Wormald PJ. The fungal microbiome in chronic rhinosinusitis: richness, diversity, postoperative changes and patient outcomes. Int Forum Allergy Rhinol (2014) 4(4):259–65. 10.1002/alr.21297 24500871

[224] Boix-Amorós A Martinez-Costa C Querol A Collado MC Mira A. Multiple approaches detect the presence of fungi in human breastmilk samples from healthy mothers. Sci Rep (2017) 7:13016. 10.1038/s41598-017-13270-x 29026146 PMC5638952

[225] Alonso R Pisa D Marina AI Morato E Rábano A Rodal I Evidence for fungal infection in cerebrospinal fluid and brain tissue from patients with amyotrophic lateral sclerosis. Int J Biol Sci (2015) 11(5):546. 10.7150/ijbs.11084 25892962 PMC4400386

[226] Alonso R Pisa D Marina AI Morato E Rábano A Carrasco L. Fungal infection in patients with Alzheimer’s disease. J Alzheimers Dis (2014) 41(1):301–11. 10.3233/JAD-132681 24614898

[227] van Woerden HC Gregory C Brown R Marchesi JR Hoogendoorn B Matthews IP. Differences in fungi present in induced sputum samples from asthma patients and non-atopic controls: a community based case control study. BMC Infect Dis (2013) 13(1):69. 10.1186/1471-2334-13-69 23384395 PMC3570489

[228] Cafarchia C Dell’Aquila M Capelli G Minoia P Otranto D Role of β-endorphin on phospholipase production in *Malassezia pachydermatis* in dogs: new insights into the pathogenesis of this yeast. Med Mycol (2007) 45(1):11–5. 10.1080/13693780600962718 17325939

[229] Cafarchia C Dell’Aquila M Traversa D Albrizio M Guaricci A De Santis T Expression of the µ-opioid receptor on *Malassezia pachydermatis* and its effect in modulating phospholipase production. Med Mycol (2010) 48(1):73–8. 10.3109/13693780902718347 19225979

[230] Honnavar P Chakrabarti A Prasad GS Singh P Dogra S Rudramurthy SM. β-Endorphin enhances the phospholipase activity of the dandruff causing fungi *Malassezia globosa* and *Malassezia restricta*. Med Mycol (2017) 55(2):150–4. 10.1093/mmy/myw058 27497434

[231] Davidson A Vermesh M Paulson RJ Graczykowski JW Lobo RA Presence of immunoreactive β-endorphin and calcitonin in human seminal plasma, and their relation to sperm physiology. Fertil Steril (1989) 51(5):878–80. 10.1016/S0015-0282(16)60684-2 2523323

[232] Shahed AR Shoskes DA Correlation of β-endorphin and prostaglandin E2 levels in prostatic fluid of patients with chronic prostatitis with diagnosis and treatment response. J Urol (2001) 166(5):1738–41. 10.1016/S0022-5347(05)65664-9 11586213

[233] Quartuccio M Immunoreactive beta-endorphin in the genital tract and seminal plasma of the bull. Reprod Domest Anim (2004) 39(4):264 10.1111/J.1439-0531.2004.498_1.X

[234] Soares RC Zani MB Arruda ACBB de Arruda LHF Paulino LC. *Malassezia* intra-specific diversity and potentially new species in the skin microbiota from Brazilian healthy subjects and seborrheic dermatitis patients. PLoS One (2015) 10(2):e0117921. 10.1371/journal.pone.0117921 25695430 PMC4335070

